# Integrated cascade antioxidant nanozymes-Cu_5.4_O@CNDs combat acute liver injury by regulating retinol metabolism

**DOI:** 10.7150/thno.106811

**Published:** 2025-04-21

**Authors:** Jiayu Chen, Yujie Zhang, Zhichao Deng, Yuanyuan Zhu, Chenxi Xu, Bowen Gao, Wenlong Wang, Jie Xiao, Zhengtao Xiao, Mingzhen Zhang, Kangsheng Tu

**Affiliations:** 1 Department of Hepatobiliary Surgery, The First Affiliated Hospital of Xi'an Jiaotong University, Xi'an, Shaanxi, 710061, China.; 2 School of Basic Medical Sciences, Xi'an Jiaotong University, Xi'an, Shaanxi, 710061, China.; 3 Guangdong Provincial Key Laboratory of Nutraceuticals and Functional Foods, College of Food Science, South China Agricultural University, Guangzhou, Guangdong 510642, China.

**Keywords:** C-dots nanozymes, Cu_5.4_O nanoparticles, Reactive oxygen species, Hepatic ischemia-reperfusion injury, Acute liver injury

## Abstract

**Background:** Acute liver failure (ALF) represents a critical medical condition marked by the abrupt onset of hepatocyte damage, commonly induced by etiological factors such as hepatic ischemia/reperfusion injury (HIRI) and drug-induced hepatotoxicity. Across various types of liver injury, oxidative stress, heightened inflammatory responses, and dysregulated hepatic retinol metabolism are pivotal contributors, particularly in the context of excessive reactive oxygen species (ROS).

**Methods:** C-dots were combined with Cu_5.4_O USNPs to synthesize a cost-effective nanozyme, Cu_5.4_O@CNDs, which mimics the activity of cascade enzymes. The *in vitro* evaluation demonstrated the ROS scavenging and anti-inflammatory capacity of Cu_5.4_O@CNDs. The therapeutic potential of Cu_5.4_O@CNDs was evaluated *in vivo* using mouse models of hepatic ischemia/reperfusion injury and LPS/D-GalN induced hepatitis, with transcriptome analysis conducted to clarify the mechanism underlying hepatoprotection.

**Results:** The Cu_5.4_O@CNDs demonstrated superoxide dismutase (SOD) and catalase (CAT) enzyme activities, as well as hydroxyl radical (·OH) scavenging capabilities, effectively mitigating ROS *in vitro*. Furthermore, the Cu_5.4_O@CNDs exhibited remarkable targeting efficacy towards inflammation cells induced by H_2_O_2_ and hepatic tissues in murine models of hepatitis, alongside exhibiting favorable biocompatibility in both *in vitro* and *in vivo* settings. Moreover, it has been demonstrated that Cu_5.4_O@CNDs effectively scavenged ROS, thereby enhancing cell survival *in vitro*. Additionally, Cu_5.4_O@CNDs exhibited significant therapeutic efficacy in mice models of HIRI and lipopolysaccharide-induced acute lung injury (LPS-ALI). This efficacy was achieved through the modulation of the ROS response and hepatic inflammatory network, as well as the amelioration of disruptions in hepatic retinol metabolism.

**Conclusions:** In summary, this study demonstrates that Cu_5.4_O@CNDs exhibit significant potential for the treatment of various acute liver injury conditions, suggesting their promise as an intervention strategy for clinical application.

## Introduction

Acute liver failure (ALF) is a clinical syndrome characterized by extensive hepatocellular necrosis, hepatic hypoplasia, and multiorgan dysfunction 1, 2. Clinical symptoms of ALF usually include hepatic dysfunction, abnormalities in liver biochemical indices, and coagulation dysfunction 3. Despite the relatively low morbidity rate, up to half of all cases may present with multiorgan failure and death, with a mortality rate of up to 30%. Consequently, there is an urgent requirement to devise effective therapeutic strategies addressing the causes of acute liver failure 4, 5.

The etiology of ALF includes hepatic ischemia-reperfusion injury (HIRI), viral infections, autoimmune hepatitis, and various other triggers of acute liver injury (ALI) 6. Several recent studies suggested that high levels of oxidative stress and inflammatory responses *in vivo* played a vital role in all types of liver injury, with reactive oxygen species (ROS) being critical 7, 8. Hepatocytes are rich in mitochondria. Disruption of the mitochondrial electron transport chain by hepatotoxic compounds, their active metabolites, and factors like hypoxia and reoxidation leads to excessive ROS production 9. Due to its powerful oxidizing properties, ROS are important inflammatory mediators and cause cellular damage at high concentrations. The main reactive oxygen species (ROS) are superoxide anion radical (O_2_^•-^), hydroxyl radical (·OH), and hydrogen peroxide (H_2_O_2_) 10. In addition, inflammation is triggered by hepatic injury, followed by the derivation of ROS mainly from activated inflammatory cells and hepatic sinusoidal endothelial cells (LSECs), which further dysregulate hepatic redox homeostasis 10, 11, creating a vicious cycle of continuous stimulation. The liver plays a crucial role in metabolism and detoxification. Numerous enzymes in the liver have overlapping substrate specificities and are typically classified as phase I (oxidative) or phase II (conjugative) drug-metabolizing enzymes. The cytochrome P450 (CYP) superfamily accounts for nearly 90% of phase I metabolic processes 12, 13. In addition, hepatic stellate cells (HSCs) store 50%-95% of human vitamin A 14, which consists of a series of retinol compounds, including retinol, retinoic acid, and retinaldehyde 15. CYP450 enzymes play an essential role in retinol metabolism, mainly involving the CYP1, CYP2C, CYP3A, and CYP26 families 16. The current study suggests that regulation of retinol metabolic homeostasis is a defining feature of hepatic stellate cells (HSCs) in both healthy and injured livers 14. Targeting excess ROS to alleviate oxidative stress, regulate inflammation, inhibit persistent stimuli, and stabilize retinol metabolism could effectively treat acute liver injury.

A robust intracellular antioxidant enzyme system, including superoxide dismutase (SOD), catalase (CAT), and peroxidase (POD), is recognized for its ability to scavenge ROS 11, 17. However, this system is insufficient to address liver injury effectively 18, 19. Antioxidant nanozymes, for example, noble metal (Au, Pt, Pd, etc.) nanoparticles, metal oxide (CeO_2_, Mn_3_O_4_, Co_3_O_4_, etc.) 20-24, synthesized from nanomaterials, are promising therapeutic agents due to their multifunctionality, including diverse antioxidant enzyme activities, catalytic activity modulation, and liver-targeted delivery through structural, compositional, and size adjustments. Our research indicates that carbon dots (C-dots) nanozymes possess SOD-like activity, effectively scavenging free radicals and offering therapeutic advantages for hepatic ischemia/reperfusion (I/R) injury 25. However, C-dots, which have only one type of enzymatic activity and generate toxic H_2_O_2_ after scavenging O_2_^•-^, still have not completely solved the problem of reactive oxygen overload. It has been shown that the coupling of noble metal nanoparticles with carbon materials allows hybridized nanozymes to achieve higher catalytic activity due to charge transfer at the metal-carbon interface and facilitated adsorption on the carbon substrate 26. For example, our previous study reported that Pt@CNDs nanocomposites have higher SOD and CAT catalytic activities than individual CNDs and PtNPs 27. Cu is an essential trace element that plays an important role in many enzymes such as tyrosinase and Cu - Zn SOD. Therefore, Cu-based nanomaterials are often developed as nanozymes with the ability to scavenge reactive oxygen species. For example, Cu NPs have excellent catalytic activity and can scavenge H_2_O_2_ and O_2_^•-^, but cannot eliminate ·OH simultaneously 28. Cu_2_O NPs have good catalytic activity to inactivate H_2_O_2_ or ·OH, thus partially mimicking peroxidase 29. Thus, Cu_5.4_O USNPs are designed to achieve both broader-spectrum enzyme catalytic performance and antioxidant activity by combining Cu_2_O and Cu nanocrystals 30. It was shown that the synergistic catalytic activity of Cu^0^ and Cu^1+^ arises from matching the rates of activation of the reactants, i.e., the rate of activation of H_2_ by Cu^0^ and the rate of activation of carbonyl-containing reactants by Cu^1+^ 31. Very interestingly, in our previous studies, we found that the hydroxyl group on the surface of CNDs binds to O_2_^•-^, which can serve as a catalytic binding site, and the carbonyl group serves as the center of its catalytic activity, i.e., the SOD-like enzyme activity of CNDs is dependent on the coupling of the carbonyl group on its surface with the π- system. Therefore, combining these two nanozymes to synthesize hybrid nanozymes can have the advantages of both high catalytic activity and SOD-CAT cascade nanozymes, and the raw materials are more economical and stable.

**Scheme 1:**
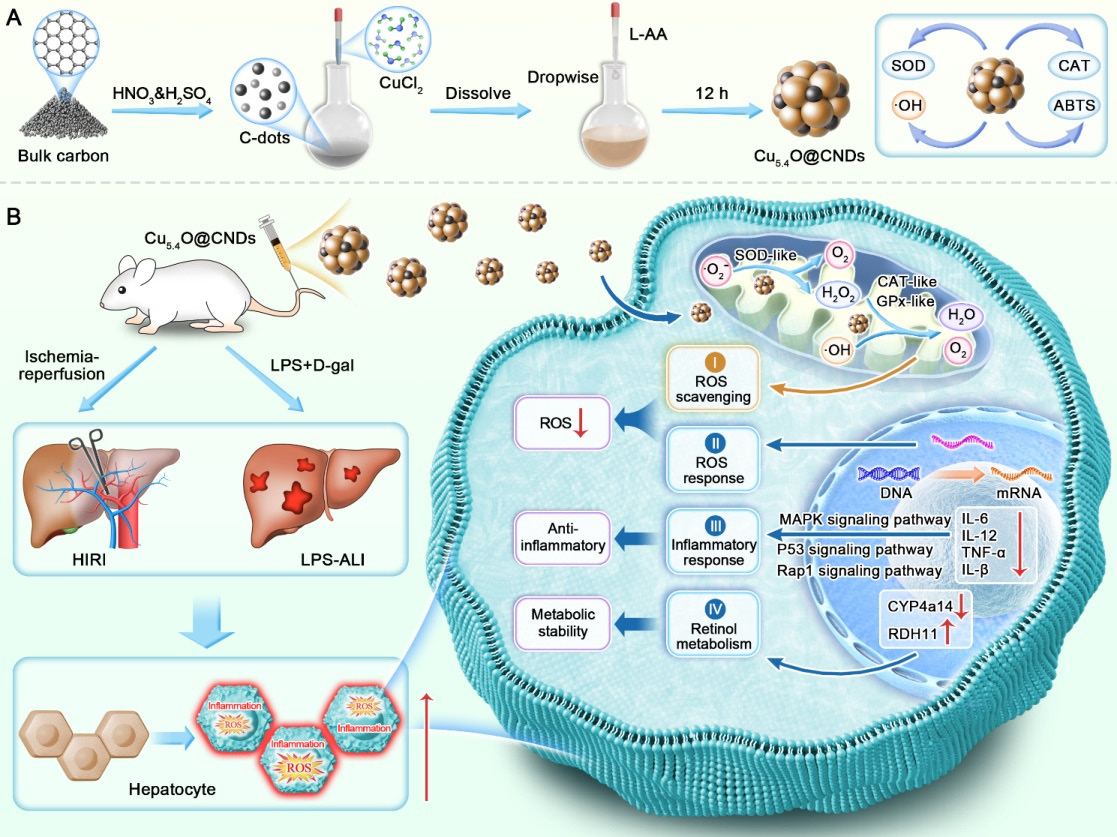
** Schematic representation of Cu_5.4_O@CNDs for the treatment of HIRI and LPS-ALI diseases.** (A) Synthesis route of Cu_5.4_O@CNDs with multiple enzymatic activities. (B) Cu_5.4_O@CNDs were injected into mice via the tail vein to scavenge excess ROS as free radical scavengers and to treat various acute liver injury diseases through anti-inflammatory, antioxidant, and regulation of retinol metabolism.

This study synthesized Cu_5.4_O@CNDs with cascade mimetic enzyme activity, which protected the liver from ROS-induced stress and inflammation and improved retinol metabolism disorders, resulting in effective therapeutic outcomes **(Scheme 1)**. It demonstrated that Cu_5.4_O@CNDs could enhance the cellular status and survival of THLE-2 and Raw264.7 cells under oxidative stress and inflammatory conditions *in vitro*. In models of HIRI and LPS-induced ALI, the administration of Cu_5.4_O@CNDs effectively scavenged ROS, reduced the expression of inflammatory cytokines, and exhibited significant therapeutic effects. Transcriptome sequencing revealed that Cu_5.4_O@CNDs provide hepatoprotection by modulating the ROS response and hepatic inflammatory network, disrupting the hepatic retinol metabolism pathway, and inhibiting apoptosis. The study highlights Cu_5.4_O@CNDs as a promising intervention for acute liver injury, supporting its potential for clinical application.

## Materials and methods

### Synthesis of Cu_5.4_O@CNDs

The C-dots were synthesized following the previously reported methodology 25. Initially, 0.5 g of activated carbon was introduced into a boiling 50 mL mixed acid solution (:=1:1) and maintained for 1.5 h. After cooling to room temperature, the C-dots samples were neutralized using a NaHCO_3_ solution. The neutralized solution was filtered using a 0.22 μm aqueous membrane and dialyzed 4-5 times daily for a week. The dialyzed C-dots solution was further filtered through a 0.22 μm aqueous membrane to remove any remaining insoluble materials. The filtrate was ultrafiltered with a 100 kDa molecular retention ultrafiltration tube. After separation, C-dots were concentrated and lyophilized for utilization in the subsequent experiments.

The Cu_5.4_O USNPs were synthesized according to the methodology reported previously. CuCl_2_ powders (10 mM) were dissolved in 50 mL of deionized water and stirred magnetically in an oil bath at 80 °C for 10 min. The CuCl_2_ solution was incrementally combined with a 50 mL aqueous solution of L-ascorbic acid (100 mM). After that, a NaOH solution (1 M) was used to bring the solution's pH down to 8.0-9.0. The mixture was continuously stirred for 12 h at 80 °C. Centrifugation (6,500 × g, 15 min) was used to remove the bigger aggregates following the reaction. The supernatant was dialyzed against water using a 10,000 Da molecular weight cutoff for 2 days to eliminate small molecules 30, 32.

According to the previous report, the synthesis pathway of Cu_5.4_O@CNDs has some modifications. Initially, 15 mM CuCl_2_ powder was dissolved in a 50 mL C-dots aqueous solution (3 mg/mL) and stirred magnetically at 80 °C for 10 min in an oil bath. Subsequently, a 300 mM L-AA aqueous solution (50 mL) was gradually added to the resulting CuCl_2_ solution. Afterward, the solution's pH was then brought down to 7.0-8.0 with a 1 M NaOH solution. For 10 h, the mixture was continuously stirred and maintained at 80 °C. Post-reaction, larger aggregates were eliminated via centrifugation at 6,577 × g for 15 min. After dialyzing against water for 2 days (Mw cutoff: 3,500 Da), the supernatant was lyophilized in preparation for further experiments. Meanwhile, CNDs-A, CNDs-B, Re-CNDs-A, CNDs-Cy5.5 were used to replace CNDs to obtain Cu_5.4_O@CNDs-A, Cu_5.4_O@CNDs-B, Cu_5.4_O@Re-CNDs-A or Cu_5.4_O@CNDs-Cy5.5.

### The SOD-like activity of C-dots, Cu_5.4_O USNPs, Cu_5.4_O@CNDs

To evaluate the SOD-like activity, a commercial SOD assay kit was used, in which the reaction substrate WST reacted with O_2_^•-^ to generate a water-soluble formazan that exhibited a distinctive absorption peak at 450 nm. When the disproportionation of O_2_^•-^ was impeded, indicating the presence of SOD-like activity in the test sample, the enzymatic activity of SOD was determined through colorimetric analysis of the WST product.

### The O_2_^•-^ scavenging activity of C-dots, Cu_5.4_O USNPs, Cu_5.4_O@CNDs

The scavenging activity of O_2_^•-^ was assessed using NBT reduction, a method that produces a blue-colored formazan in the presence of O_2_^•-^. When C-dots with SOD-like activity are present, they competitively scavenge O_2_^•-^, resulting in a lighter or absent blue color. Samples with six concentrations (0-200 μg/mL) were combined with NBT (0.05 mM), L-met (13 mM), and riboflavin (20 μM) in a PBS buffer (pH 7.4, 25 mM) for investigation. The mixture was then irradiated by LED for 5 min.

The scavenging activity of O_2_^•-^ was assessed using ESR assay. Hypoxanthine/xanthine oxidase (HYP/XOD) produces O_2_^•-^ by mixing DMPO, DTPA, HYP, XOD, and Cu_5.4_O@CNDs in PBS (pH = 7.3), and the ESR spectra of BMPO/⋅OOH were recorded after 2 min.

### The CAT-like activity of C-dots, Cu_5.4_O USNPs, Cu_5.4_O@CNDs

O_2_ production from H_2_O_2_ scavenging by different materials was determined using dissolved oxygen. A reaction system with a final volume of 15 mL of ultrapure water containing 60 μL of 30% H_2_O_2_ and various concentrations of different materials was then kinetically measured using a dissolved oxygen meter (JPSJ-605F, Ray Magnet) for 15 min.

### The H_2_O_2_ scavenging activity of C-dots, Cu_5.4_O USNPs, Cu_5.4_O@CNDs

The Hydrogen Peroxide Detection Kit was used to evaluate the H_2_O_2_ scavenging capacity of C-dots, Cu_5.4_O USNPs, and Cu_5.4_O@CNDs. An absorbance peak at 405 nm is observed when ammonium molybdate reacts with H_2_O_2_. Samples were incubated at 37 °C for 2 h with different concentrations of 2 mM H_2_O_2_, following the manufacturer's instructions.

The scavenging activity of H_2_O_2_ was assessed using ESR assay. Different 100 µg/mL materials catalyzed the degradation of H_2_O_2_ to generate O_2_. The ESR spin probe CTPO was employed to capture the O_2_, and the ESR spectra of CTPO exhibited a proton hyperfine structure in a control nitrogen-saturated solution. Increasing O_2_ concentration elevated the collision frequency between oxygen and nitrogen-oxygen radicals, leading to a broadened ESR triplet state spectrum and reduced resolution of the proton hyperfine structure.

### The ⋅OH scavenging activity of C-dots, Cu_5.4_O USNPs, Cu_5.4_O@CNDs

The ⋅OH scavenging activity was evaluated via the TMB assay, utilizing solutions of 10 µM FeCl_2_, 50 µM H_2_O_2_, and 300 µM TMB, all prepared with ultrapure water. The three solutions were then mixed homogeneously. The mixtures were prepared by thoroughly combining with sample solutions ranging from 0 to 200 µg/mL and allowed to react for 30 min at room temperature, followed by absorbance measurement at 645 nm.

The scavenging activity of ⋅OH was assessed using an ESR assay. The Fenton reaction produced ⋅OH by combining 100 mM DMPO, 1.0 mM FeSO_4_, 1.0 mM H_2_O_2_, and 100 µg/mL Cu_5.4_O@CNDs in ddH_2_O.

### ABTS^•+^ radical scavenging activity of C-dots, Cu_5.4_O USNPs, Cu_5.4_O@CNDs

Following instructions, the ABTS^•+^ radical scavenging capacity of C-dots, Cu_5.4_O USNPs, and Cu_5.4_O@CNDs was assessed using the T-AOC analysis kit (S0119, Beyotime). The ABTS^•+^ and oxidant solution were combined in a 1:1 volume ratio to create a fresh working fluid, which was then stored at ambient temperature and shielded from light for 12-16 h before utilization. The working mix was subsequently diluted according to the kit instructions, and the samples were analyzed at various concentrations of different materials (0-200 μg/mL) as specified.

### Cell culture

THLE-2 cells were maintained at 37 °C in a humidified incubator with 5% CO_2_ using a cell-specific medium. Raw 264.7 cells were maintained in DMEM (Gibco) with 10% fetal bovine serum and antibiotics at 37°C in a 5% CO_2_ humidified incubator.

### Cell targeting

Preparation of Cy5.5-loaded Cu_5.4_O@CNDs for cellular uptake studies. THLE-2 and Raw 264.7 cells were each incubated for 24 h before being cocultured with Cu_5.4_O@CNDs for an additional 4 h. Subsequently, the cells were fixed, stained with DAPI, and washed multiple times. The cells were then imaged with a fluorescence microscope.

### *In vitro* oxidative stress and inflammation model

To examine the capacity of Cu_5.4_O@CNDs to scavenge ROS in cells, firstly, the cells underwent 12 h of nutrient deprivation. Then, an oxidative stress model was established *in vitro*. After a 24 h incubation using the specified cell culture method, cells were treated with Cu_5.4_O@CNDs at concentrations ranging from 0 to 80 µg/mL for 8-12 h. They were then exposed to either 1 mM H_2_O_2_ or 1 µg/mL LPS and incubated at 37 °C for an additional 1 or 8 h.

### *In vitro* ROS scavenging using Cu_5.4_O@CNDs nanozymes

After the treatment, the cells were gently rinsed with a serum-free medium. Cells were incubated in darkness at 37 °C for 30 min in a serum-free medium containing 10 μM of 2,7-dichlorofluorescein diacetate (DCFH-DA) and dihydroethidium (DHE). Following incubation, cells underwent three washes to eliminate surplus DCFH-DA and DHE probes. Then, the cells were imaged using fluorescence microscopy, and flow cytometry analysis was performed to quantify intracellular ROS levels. Furthermore, the treated cells were stained with 5,5′,6,6′-Tetrachloro-1,1′,3,3′-tetraethyl-imidacarbocyanine iodide (JC-1) for 30 min. Fluorescence microscopy was employed to assess cell protection by Cu_5.4_O@CNDs.

### *In vitro* anti-inflammatory using Cu_5.4_O@CNDs nanozymes

After the incubation of Raw 264.7, the cells were separately incubated with Cu_5.4_O@CNDs for 8 h, followed by overnight incubation with 1 μg/mL lipopolysaccharide (LPS). Cells were stained with CD 80 marker cells followed by flow cytometry to assess the extent of macrophage polarization. THLE-2 and Raw 264.7 cells were incubated in a 6-well plate and stained with JC-1 for 30 min. Following incubation, the cells underwent three washes to remove any residual JC-1 probe. Fluorescence microscopy was employed to assess cell protection by Cu_5.4_O@CNDs. THLE-2 and Raw 264.7 cells were incubated Cu_5.4_O@CNDs for 8 h, followed by an overnight incubation with 1 μg/mL LPS. Cells were collected, RNA was extracted, and mRNA expression levels of inflammatory factors IL-1β, IL-6, IL-12, and TNF-α were measured using an rt-QPCR kit. The primer sequences of related genes are shown in **Table S1**.

### *In vivo* biodistribution

Initially, Cy5.5-containing nanosystems were prepared at a dose of 0.5 mg/kg. Subsequently, BALB/C mice were randomly divided into two groups: healthy mice and those with hepatitis. Each group was randomized into two groups: the Cy5.5-labeled group, and the Cu_5.4_O@CNDs group. The *in vivo* distribution of nanomaterials was assessed using a near-infrared small animal imager within 0-4 h following tail vein injection. At the endpoint, mice were euthanized, and their heart, liver, spleen, lung, and kidney tissues were collected for *ex vivo* fluorescence imaging.

### Biocompatibility of C-dots nanozymes

Cell viability was evaluated via the MTT assay after co-incubation with Cu_5.4_O@CNDs nanozymes at concentrations ranging from 0 to 100 μg/mL for 12 and 24 h, following the specified cell culture protocol. The *in vivo* biocompatibility of Cu_5.4_O@CNDs was assessed by intravenously administering 0.5 mg/kg of Cu_5.4_O@CNDs to BALB/c mice. Control mice were injected with PBS. Controls were mice injected with PBS. Blood samples were collected for complete blood cell analysis and serum biochemical tests after 1 day and 7 consecutive days of injection. The mice were euthanized, and their major organs were collected for histological examination using H&E staining.

### HIRI model in mice

Male BALB/c mice aged 6-8 weeks were selected 25. Anesthesia was administered using a small animal anesthesia machine. A midline abdominal incision was made to expose the liver, and noninvasive vascular clips were used to occlude the left branch of the hepatic artery, the left hepatic duct, and the portal vein. The left and middle lobes of the liver appeared white, indicating 70% partial ischemia. After 1 h, the clamps were removed, and the liver tissue became red and moist, this confirms that circulation has been restored. The abdominal incision was then sutured. Mice were euthanized 24 h after blood flow was restored.

### *In vivo* therapeutic effect of Cu_5.4_O@CNDs on HIRI

BALB/c mice with an established HIRI model were randomly assigned to six groups (n = 5): Sham-operated (control), HIRI, and Cu_5.4_O@CND therapeutic doses of 0.1 mg/kg, 0.5 mg/kg, 1.0 mg/kg, and 2.5 mg/kg. Mice weight changes in each group were monitored 24 h post-treatment. 24 h after injection, mice were euthanized, and blood samples were collected to quantify the levels of albumin transaminase (AST) and alanine transaminase (ALT). Liver tissues were collected and homogenized to determine the transcription levels of pro-inflammatory factors. The primer sequences of related genes are shown in **Table S1**. At the same time, a portion of the liver tissue was paraffin-embedded for H&E and TUNEL staining. The other portion of liver tissue was freeze-embedded and cryosectioned. The frozen liver sections were stained with DAPI and DCFH/DHE. Fluorescence microscopy is used to qualitatively evaluate ROS levels in liver tissue sections.

### LPS-ALI model in mice

Male BALB/c mice, aged 6-8 weeks, were selected and underwent a 15 h period of food and water deprivation. They were injected intraperitoneally with 30 μg/mL LPS, 200 mg/kg D-gal, and euthanized after 12 h.

### *In vivo* therapeutic effect of Cu_5.4_O@CNDs on LPS-ALI

BALB/c mice with an established LPS-ALI model were randomly assigned to three groups (n = 5): a PBS control group, an LPS-ALI group, and a treatment group receiving 0.5 mg/kg of Cu_5.4_O@CNDs. Mice were executed after 12 h, serum was taken, liver tissue was removed, and the same validation experiments as in the HIRI model were performed. The primer sequences of related genes are shown in **Table S1**.

### Transcriptome analysis

HIRI and LPS-ALI model mice were divided into three groups: Con, modeling group, and treatment group, respectively. Liver tissues were collected from mice post-experiment and kept on ice. RNA was isolated from liver tissue, and its purity and concentration were assessed using a NanoDrop 2000 spectrophotometer. Sequencing libraries were constructed using high-quality RNA samples meeting the criteria: OD_260/280_ = 1.8-2.2, RIN ≥ 6.5, 28S: 18S ≥ 1.0, > 2 μg. The library underwent bipartite sequencing using the Illumina NovaseqTM 6000 (LC Bio-Technology CO., Ltd., Hangzhou, China), following standard procedures, with a sequencing mode of PE150.

### Western blotting

Liver tissues were lysed using RIPA buffer (WB3100, NCM Biotech, China) supplemented with a protease inhibitor cocktail (P001, NCM Biotech, China). Protein concentrations were determined using a BCA protein assay kit (WB6501, NCM Biotech, China). Equal amounts of protein were then separated by SDS-PAGE and transferred onto PVDF membranes. The membranes were blocked with 5% milk in TBST for 1 h, followed by incubation with primary antibodies overnight at 4 °C. After 1 h of incubation with secondary antibodies, the membranes were visualized using an enhanced chemiluminescence system, and the images were quantified using ImageJ software. Details of the antibodies and their dilutions are provided in **Table S2**.

### Statistical analysis

The GraphPad Prism 9 software was used to analyze data. A two-tailed Student's t-test was employed for comparing two groups, while one-way ANOVA was utilized for comparisons involving multiple groups. Statistical significance was assessed with thresholds: ns (not significant), *P < 0.05, **P < 0.01, ***P < 0.001, and ****P < 0.0001.

## Results and Discussion

### Transcriptomic sequencing of HIRI and LPS-ALI to analyze the potential pathogenesis

**Figure 1:**
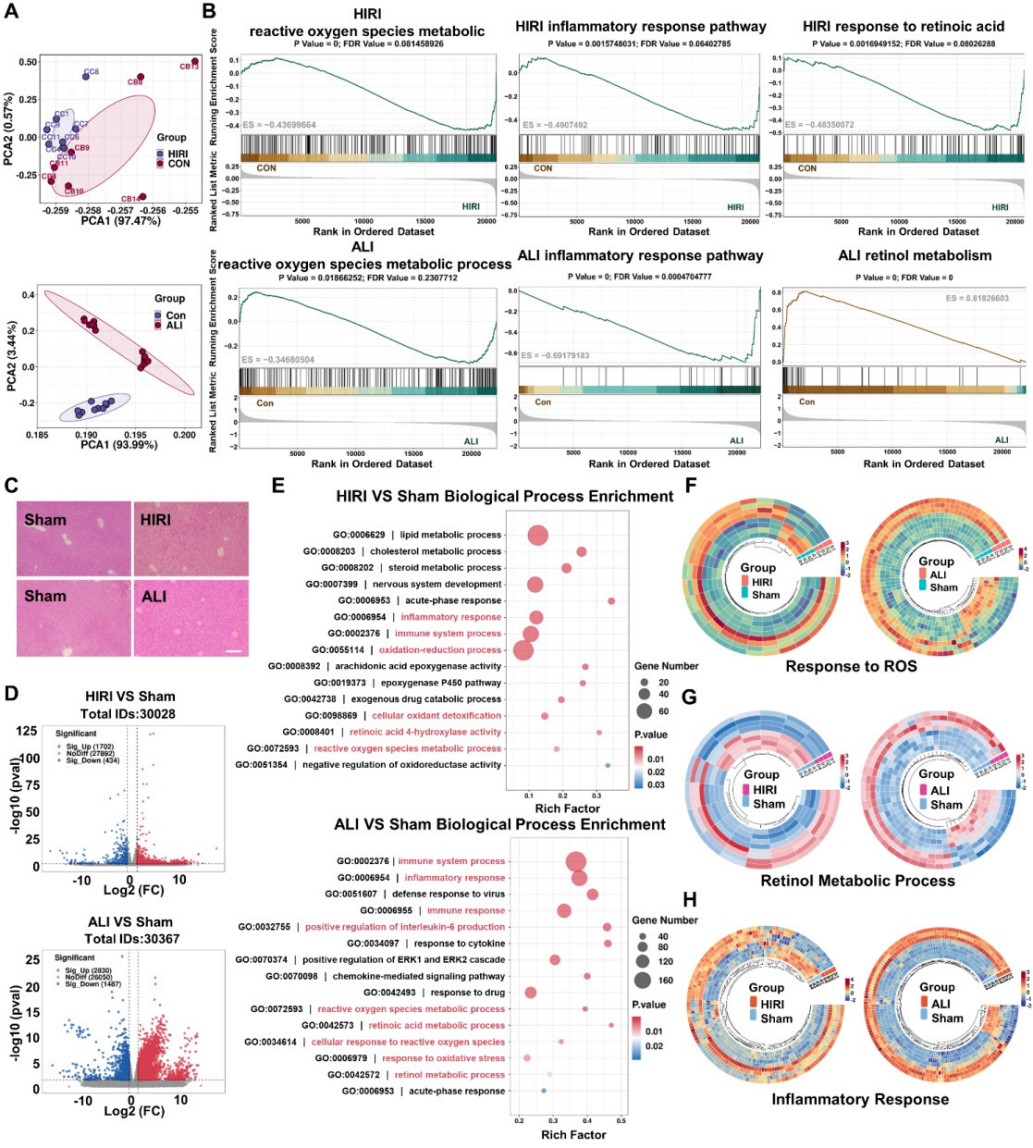
** Transcriptomic sequencing of HIRI and LPS-ALI to analyze potential pathogenesis.** (A) PCA analysis was done using the GSE112713 database and the GSE38941 database. (B) GSEA analysis of ROS metabolic process, inflammatory response pathway, and retinol metabolism was done using the GSE112713 database and GSE38941 database. (C) H&E staining of Con, HIRI, and LPS-ALI. (D) Volcano plots showed the identified upregulated and downregulated genes by Cu_5.4_O@CNDs. (E) GO (Biological Process) analysis. The 15 most significantly enriched pathways are shown. (F) Differential gene heat maps associated with Response to ROS (fold change ≥ 2 and P < 0.01). (H) Differential gene heat maps associated with Retinol metabolic process (fold change ≥ 2 and P < 0.01). (G) Differential gene heat maps associated with Inflammatory response (fold change ≥ 2 and P < 0.01).

The RNA sequencing of acute liver injury disease by the GEO database was analyzed to find an effective treatment for acute liver injury disease. Sample correlation analysis between patients and healthy controls was performed, and the results of PCA analysis suggested that the screened GSE112713 database and GSE38941 database had good intragroup clustering, and the gene expression differences between the healthy control group and the disease group were significant enough to allow for subsequent analysis **(Figure 1A)**. GSEA analysis of the two databases separately showed that the ROS metabolic process 8, 20, inflammatory response pathway 7, 33, and retinol metabolism 12 were significantly abnormal in patients with HIRI and ALI, which might be an effective therapeutic target **(Figure 1B)**. Meanwhile, to verify the consistency of the pathogenesis of acute liver injury at the mice level with that in humans, mice models of HIRI and LPS-ALI were established, and the H&E results suggested that apparent inflammatory cell infiltration and tissue necrosis could be observed **(Figure 1C)**. Tissue RNA sequencing revealed statistically significant gene expression differences between control and modeling groups in HIRI and LPS-ALI models, confirming successful disease model establishment for further analysis **(Figure 1D)**. Then, based on the sequencing results, GO analysis was based on the differential genes of the two disease models (HIRI and LPS-ALI), and the top 15 metabolic processes were listed. The results suggested that both disease processes were highly correlated with inflammatory responses, ROS metabolic processes, oxidative stress, and retinol metabolism pathways **(Figure 1E)**. To further clarify the differential gene expression profiles of our pathways of interest, a heatmap analysis of the differential genes related to the ROS metabolic process, inflammatory response pathway, and retinol metabolic pathway was performed. Significant differences in gene expression between the disease models (HIRI and LPS-ALI) and control mice are evident within the same metabolic pathway** (Figure 1F-H)**. The above results demonstrated that ROS metabolic processes, inflammatory response pathways, and retinol metabolism were highly relevant in the pathogenesis of two acute liver injury diseases, HIRI and LPS-ALI.

### Synthesis and characterization of C-dots, Cu_5.4_O USNPs, Cu_5.4_O@CNDs

**Figure 2:**
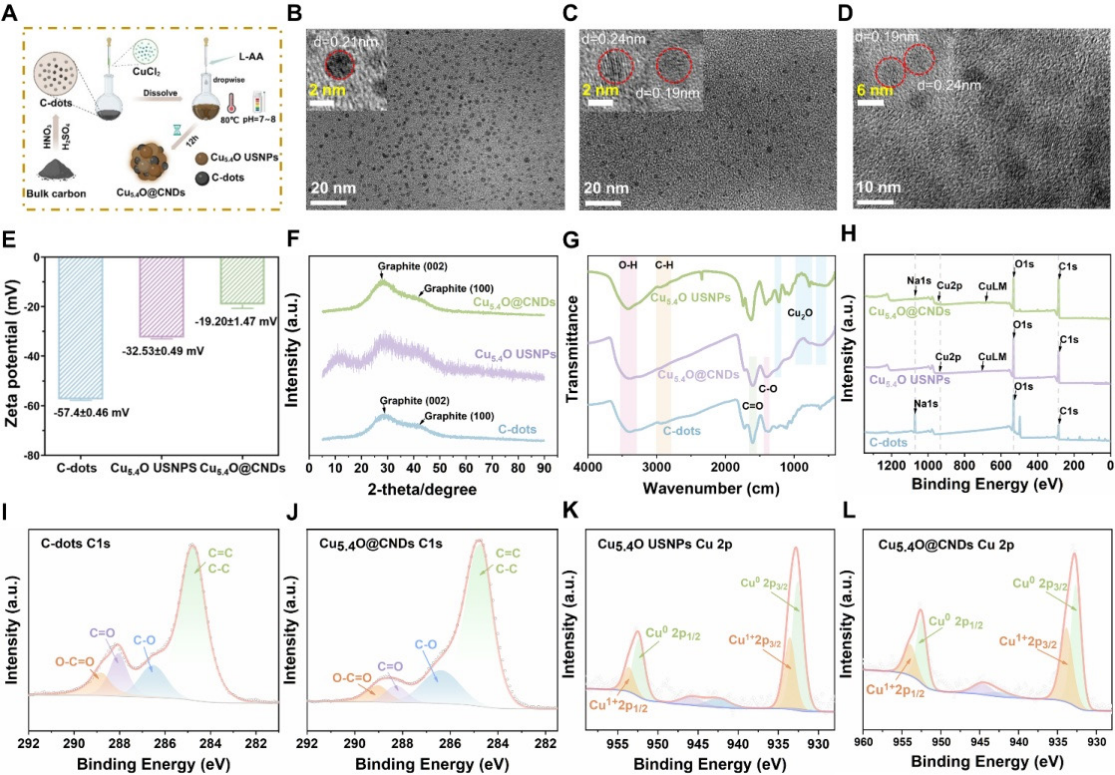
** Synthesis and characterization of C-dots, Cu_5.4_O USNPs, Cu_5.4_O@CNDs.** (A) Synthesis path diagram of C-dots, Cu_5.4_O USNPs, Cu_5.4_O@CNDs. The figure was created partially with BioRender.com. (B-D) TEM and HR-TEM images of C-dots, Cu_5.4_O USNPs, and Cu_5.4_O@CNDs. (E) Zeta potential of C-dots, Cu_5.4_O USNPs, Cu_5.4_O@CNDs. (F) XRD patterns of C-dots, Cu_5.4_O USNPs, Cu_5.4_O@CNDs. (G) FTIR spectra of C-dots, Cu_5.4_O USNPs, Cu_5.4_O@CNDs. (H) XPS patterns of C-dots, Cu_5.4_O USNPs, Cu_5.4_O@CNDs. (I-J) C 1s split-peak fitting profiles of C-dots and Cu_5.4_O@CNDs. (K-L) Cu 2p split-peak fitting profiles of Cu_5.4_O USNPs and Cu_5.4_O@CNDs.

Prior research has demonstrated that C-dots nanozymes serve as free radical scavengers and significantly ameliorate HIRI injury by influencing hepatic inflammation and retinol metabolism pathways 25. Cu_5.4_O USNPs, known for their enzyme mimicry and broad-spectrum ROS scavenging capabilities, have been shown to aid in treating various ROS-related diseases, including acute kidney and liver injuries, by regulating ROS-related genes and modulating multiple classical inflammatory pathways 30. Based on this, a cascade mimetic enzyme Cu_5.4_O@CNDs was designed by combining the two to synthesize green and economical nanozymes. Cu_5.4_O@CNDs, C-dots, and Cu_5.4_O USNPs were synthesized using a simple, efficient one-pot method** (Figure 2A)**. The optimal synthesis conditions were determined by adjusting the Cu^2+^ to L-ascorbic acid (AA) ratio, C-dots input concentration, pH, and final Cu^2+^ concentration to evaluate their impact on the catalytic activity of the resulting materials 30, 34. The catalysis activity of the synthesized Cu_5.4_O USNPs was almost the same at Cu^2+^ to AA feed ratios of 1:5, 1:10, 1:40, and 1:60. The optimal feeding ratio was 1:20, leading to a Cu^2+^ to AA molar ratio of 1:20, with a Cu^2+^ concentration of 15 mM, C-dots at 3 mg/mL, and a pH range of 7.0-8.0 **(Figure S1)**.

Transmission electron microscopy (TEM) analysis revealed that C-dots were homogeneously and monodisperse distributed, with a mean particle size of 2.39 ± 0.50 nm, as depicted in the statistical graph of the particle size distribution. The lattice of 0.21 nm corresponds to the (100) face of graphite 27 **(Figure 2B and Figure S2A)**. TEM analysis of Cu_5.4_O USNPs revealed uniformly spherical nanoparticles with an average size of 2.06 ± 0.50 nm, as depicted in the particle size distribution graph. The lattice of 0.24 nm corresponds to the (111) face of Cu_2_O, and the lattice of 0.19 nm corresponds to the (111) face of Cu(0) 35, 36 **(Figure 2C and Figure S2B)**. Cu_5.4_O USNPs and C-dots were synthesized into the polymer, with TEM analysis indicating uniformly spherical nanoparticles and an average particle size of 5.87 ± 0.94 nm. The lattice of 0.21 nm in Cu_5.4_O@CNDs could be ascribed to the (100) facet of graphite observed in C-dots, while the lattice of 0.24 nm could be ascribed to the (111) facet of Cu_2_O observed in Cu_5.4_O USNPs, which can indicate that Cu_5.4_O@CNDs was successfully synthesized by C-dots and Cu_5.4_O USNPs **(Figure 2D and Figure S2C)**. The zeta potential of C-dots was -51.7 ± 0.9 mV, and it increased to -60.47 ± 0.67 mV upon the addition of Cu_5.4_O USNP_S_, indicating the successful integration of C-dots with Cu_5.4_O USNPS **(Figure 2E)**. X-ray powder diffractograms (XRD) results of C-dots, Cu_5.4_O USNP_S_, and Cu_5.4_O@CNDs showed that the C-dots had prominent diffraction peaks at 2θ at 25-30° and 42°, and the Cu_5.4_O USNP_S_ had obvious 2θ at 30°, 42° and 50° diffraction peaks at 2θ at 25-30°, 42°, 50°. All appeared relative to C-dots and Cu_5.4_O USNP_S_, indicating the successful synthetization of Cu_5.4_O@CNDs **(Figure 2F)**. The FI-IR results showed that the characteristic peaks at 3300-3500 cm^-1^ were attributed to the O-H stretching vibration, while the absorption peaks in the range of 2870-2980 cm^-1^ were attributed to the aliphatic hydrocarbon C-H stretching vibration. The characteristic peaks at 1650-1750 cm^-1^ and 1350-1450 cm^-1^ for the C-dots and Cu_5.4_O@CNDs are attributed to the stretching vibrations of C=O and C-O, respectively. The absorption peaks at 530-622 cm^-1^, 774-1075 cm^-1^, and 1219 cm^-1^ for Cu_5.4_O USNP_S_ and Cu_5.4_O@CNDs are characteristic peaks of Cu_2_O. Therefore, the results of FI-IR experiments suggested that Cu_5.4_O@CNDs possessed the characteristic bands of both C-dots and Cu_5.4_O USNPs, which proved the successful synthesis of Cu_5.4_O@CNDs **(Figure 2G)**. X-ray photoelectron spectroscopy (XPS) maps are commonly employed to examine the elemental bonding in composites. The total XPS spectra showed the elemental peaks of Na 1s, O 1s, and C 1s in C-dots, and Cu 2p and Cu LM peaks in Cu_5.4_O USNPs, while the elemental peaks of Na 1s, Cu 2p, Cu LM, O 1s, and C 1s were detected in Cu_5.4_O@CNDs, which proved the successful integration of C-dots and Cu_5.4_O USNPS **(Figure 2H)**. C 1s fractional peak fitting maps, including graphitic carbon at 284.8 eV, alcoholic carbon at 286.6 eV, carbonyl carbon at 288.1 eV, and carboxy carbon at 288.9 eV, were detected in C-dots and Cu_5.4_O@CNDs, which showed that the characteristic peaks of C-dots appeared in Cu_5.4_O@CNDs **(Figure 2I-J)**. The Cu 2p split-peak fitting profiles of Cu_5.4_O USNPS and Cu_5.4_O@CNDs confirmed the presence of Cu^0^ and Cu^1+^ in Cu_5.4_O@CNDs **(Figure 2K-L)**. The above results suggested that the Cu_5.4_O@CNDs were successfully synthesized and retained the respective characteristic structures and characterization of C-dots and Cu_5.4_O USNP_S_.

### Enzymatic characterization of C-dots, Cu_5.4_O USNPs, Cu_5.4_O@CNDs

**Figure 3:**
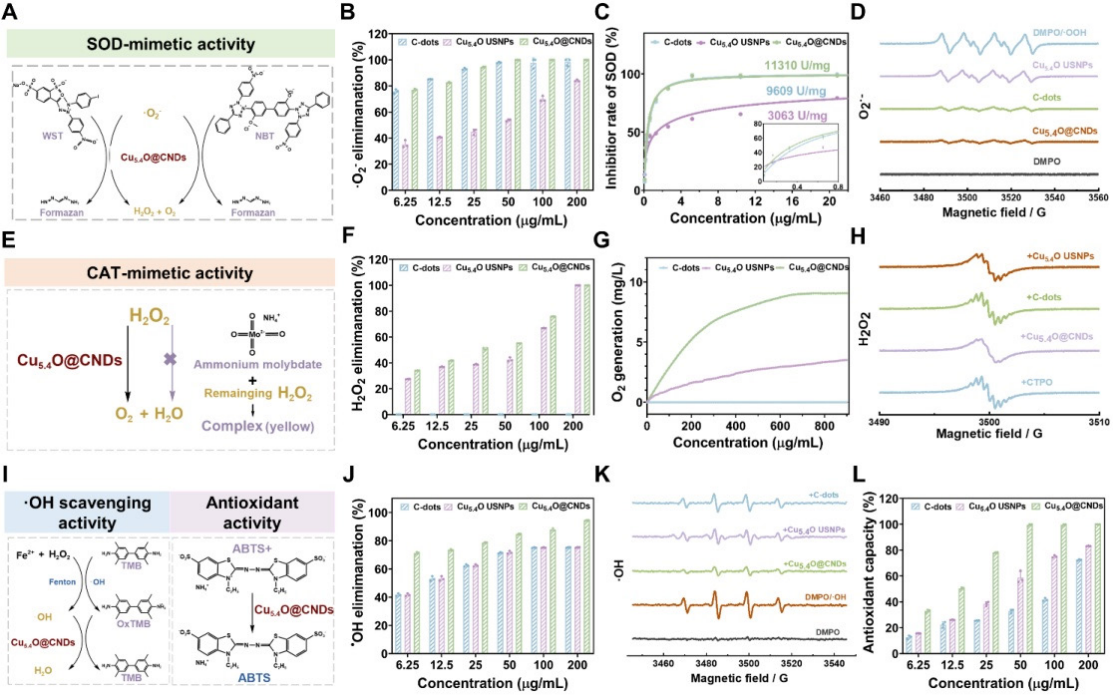
** Enzymatic characterization of C-dots, Cu_5.4_O USNPs, Cu_5.4_O@CNDs.** (A) Schematic diagram of O_2_^•-^ scavenging by SOD-like enzymes. (B) The O_2_^•-^ the scavenging capability of C-dots, Cu_5.4_O USNPs, Cu_5.4_O@CNDs. (C) The WST-1 kit evaluated the SOD-like activity of C-dots, Cu_5.4_O USNPs, and Cu_5.4_O@CNDs. (D) ESR assay for O_2_^•-^ scavenging capability of C-dots, Cu_5.4_O USNPs, Cu_5.4_O@CNDs. (E) The schematic diagram for the scavenging of H_2_O_2_ by CAT-like enzymes. (F) Ultraviolet absorption test H_2_O_2_ scavenging capability of C-dots, Cu_5.4_O USNPs, Cu_5.4_O@CNDs. (G) The dissolved oxygen level of C-dots, Cu_5.4_O USNPs, and Cu_5.4_O@CNDs reacted with H_2_O_2_ detected by an oxygen sensor. (H) ESR assay for H_2_O_2_ scavenging capability of C-dots, Cu_5.4_O USNPs, Cu_5.4_O@CNDs. (I) The schematic diagram for the scavenging of ⋅OH and ABTS^•+^ radicals. (J) The ⋅OH scavenging capability of C-dots, Cu_5.4_O USNPs, Cu_5.4_O@CNDs. (K) ESR assay for ⋅OH scavenging capability of C-dots, Cu_5.4_O USNPs, Cu_5.4_O@CNDs. (L) The ABTS^•+^ radical scavenging capability of C-dots, Cu_5.4_O USNPs, Cu_5.4_O@CNDs. Data represent means ± s.d. from three independent replicates. ns: not significant, *P < 0.05, **P < 0.01, ***P < 0.001, one-way ANOVA.

O_2_^•-^ is a well-known and common type ROS, and the level of SOD-like enzyme activity of the material can be assessed by testing its scavenging rate **(Figure 3A)**. Firstly, the NBT reduction method can indirectly detect the SOD-like enzyme activity of C-dots, Cu_5.4_O USNPs, and Cu_5.4_O@CNDs. In this system, O_2_^•-^ can make the NBT coloration produce blue formazen. Suppose the material has SOD activity that can remove O_2_^•-^. In that case, the coloration of NBT will be weaker and, at the same time, indirectly indicate that there is SOD-like enzyme activity. Under a series of concentration gradients, Cu_5.4_O@CNDs clearly showed superior O_2_^•-^ scavenging efficiency than Cu_5.4_O USNP_S_, which was basically in line with C-dots **(Figure 3B)**. The SOD-like enzyme activities of C-dots, Cu_5.4_O USNP_S_, and Cu_5.4_O@CNDs were measured using a SOD assay kit. The study found that C-dots exhibited SOD-like enzyme activity of 9609 U/mg, whereas Cu_5.4_O USNPs demonstrated an activity of 3063 U/mg. The SOD-like enzyme activity of Cu_5.4_O@CNDs was 11310 U/mg, superior to that of the other two materials described **(Figure 3C)**. In addition, the scavenging ability of Cu_5.4_O@CNDs for O_2_^•-^ was assessed by ESR, which detected the changes in the intensity of free radical signals. It showed that no ESR signal was present in DMPO alone. In contrast, a distinct signal peak appeared when both DMPO and O_2_^•-^ were present, indicating that DMPO successfully captured O_2_^•-^ and exhibited vigorous signal intensity. The addition of 100 μg/mL of C-dots, Cu_5.4_O USNP_S_, and Cu_5.4_O@CNDs significantly weakened the signal peak intensity, suggesting that all three materials can remove O_2_^•-^. Among them, the signal peaks were weakened to a greater extent after the addition of C-dots and Cu_5.4_O@CNDs, which proved that Cu_5.4_O@CNDs could effectively scavenge O_2_^•-^ and possess a good antioxidant capacity **(Figure 3D)**.

H_2_O_2_, as the product of disproportionation catalyzed by SOD, is also a kind of toxic ROS, and to achieve the purpose of cascade scavenging of ROS, H_2_O_2_ needs to be decomposed into non-toxic oxygen and water, which can be catalyzed by catalase **(Figure 3E)**. Firstly, in different concentrations of C-dots, Cu_5.4_O USNPs, and Cu_5.4_O@CNDs in solutions of H_2_O_2_ (10 mM), C-dots had no H_2_O_2_ scavenging ability, Cu_5.4_O USNPs and Cu_5.4_O@CNDs possessed concentration-dependent decreases in absorption values at 240 nm, and Cu_5.4_O@CNDs had a higher H_2_O_2_ scavenging rate than Cu_5.4_O USNPS **(Figure 3F)**. Then, the CAT activities of C-dots, Cu_5.4_O USNPs, and Cu_5.4_O@CNDs were measured by the dissolved oxygen concentration detected by a dissolved oxygen meter. The results showed that in the C-dots group, almost no O_2_ was produced, and the Cu_5.4_O@CNDs group produced twice as much O_2_ as the Cu_5.4_O USNPs group, which indicated that C-dots had no H_2_O_2_ scavenging ability. The rate of H_2_O_2_ decomposition by Cu_5.4_O@CNDs could reach about two times that of Cu_5.4_O USNPs. The oxygen production capacity of Cu_5.4_O USNPs and Cu_5.4_O@CNDs increased in a dose-dependent manner **(Figure 3G)**. In addition, the generation of O_2_ catalyzing the degradation of H_2_O_2_ was tested using ESR test materials. The ESR spin probe CTPO was employed to capture oxygen, revealing a proton hyperfine structure in a nitrogen-saturated control solution. As oxygen concentration increased, the collision frequency between oxygen and nitrogen-oxygen radicals rose, leading to a broadening of the ESR triplet state spectrum and a reduction in the resolution of the proton hyperfine structure. The results showed that the fine peak of CTPO did not change after the addition of C-dots, which verified that C-dots do not have H_2_O_2_ scavenging ability and only have SOD enzyme activity. When Cu_5.4_O USNPs and Cu_5.4_O@CNDs were added, the CTPO fine peak signals appeared attenuated. Among them, the addition of Cu_5.4_O@CNDs showed a large decrease in the CTPO fine peak signal, which proved that Cu_5.4_O@CNDs had a significantly better H_2_O_2_ scavenging ability than the other two materials **(Figure 3H)**.

·OH is another important ROS with oxidizing solid properties, so ·OH scavenging can also effectively protect cells from oxidative damage **(Figure 3I)**. The TMB method was employed to indirectly evaluate the hydroxyl radical scavenging effect using the Fenton reaction (Fe^2+^/H_2_O_2_) to produce hydroxyl radicals, which catalyzed TMB to form soluble blue products. The addition of C-dots, Cu_5.4_O USNPs, and Cu_5.4_O@CNDs altered the color, and measurements were taken using an enzyme marker to calculate the scavenging rate, achieving over 80% clearance at 25 μg/mL. Cu_5.4_O@CNDs significantly outperformed the hydroxyl radical scavenging ability of the other two materials mentioned above **(Figure 3J)**. Meanwhile, the ·OH scavenging ability was detected with the help of ESR to detect the intensity of free radical signaling. The system utilized the Fenton reaction to generate ·OH radicals, which were subsequently captured by DMPO to form DMPO/·OH spin adducts. When Fenton's reagent was mixed with DMPO, a tetra-linear eigen peak with apparent signal intensity appeared, which proved that a large amount of ·OH was generated in this mixture system. In contrast, the intensity of the tetra-linear eigen peak was reduced when C-dots, Cu_5.4_O USNPs, and Cu_5.4_O@CNDs were added. The intensity of the tetra-linear eigen peak of Cu_5.4_O@CNDs was almost reduced. The four-linear state characteristic peaks of Cu_5.4_O@CNDs nearly disappeared, indicating that Cu_5.4_O@CNDs can effectively scavenge ·OH** (Figure 3K)**.

The above experiments describe the practical scavenging ability of Cu_5.4_O@CNDs in the presence of one free radical alone. The ABTS^•+^ method was employed using a total antioxidant capacity kit to assess the radical scavenging ability of Cu_5.4_O@CNDs **(Figure 3I)**. They were diluting the master batch according to the kit's instructions and testing the samples at different concentrations according to the requirements. The results showed that C-dots, Cu_5.4_O USNPs, and Cu_5.4_O@CNDs had good total antioxidant levels, the antioxidant capacity was concentration-dependent, and the total antioxidant capacity of Cu_5.4_O@CNDs was significantly better than the others; more than 80% clearance can be achieved at 25 μg/mL **(Figure 3L)**. The above results exhibited that Cu_5.4_O@CNDs not only possessed excellent SOD and CAT activities but also were able to scavenge hydroxyl radicals, as well as possessed high antioxidant capacity, which indicated that Cu_5.4_O@CNDs were expected to treat the ROS-related injuries at cellular and animal levels.

### The mechanism of synergy effect between Cu_5.4_O USNPs and CNDs

To investigate why Cu_5.4_O@CNDs have better SOD and CAT-like enzyme activities than C-dots and Cu_5.4_O USNPs, we synthesized Cu_5.4_O@CNDs by replacing CNDs with passivating the hydroxyl, carboxyl, and carbonyl groups on the surface of CNDs, then we investigated the synergistic mechanism between Cu_5.4_O USNPs and CNDs and the catalytic mechanism of their activity. First, to investigate the contribution of hydroxyl groups on the surface of CNDs to the formation of Cu_5.4_O@CNDs, we selectively deactivated the carboxyl and hydroxyl groups using 1,3 -propanesulfonic acid ketone (PS). PS reacted with the carboxyl and hydroxyl groups on the surface of the CNDs to form an ester and an ether, respectively, as demonstrated in our previous work. Esters can be hydrolyzed under alkaline conditions, whereas ethers cannot. Hydrolysis of CNDs in 0.5 M sodium hydroxide solution (reaction with PS) yielded hydroxyl-only passivated CNDs- A **(Figure S3A)**. To investigate the contribution of carboxyl groups on the surface of CNDs to the formation of Cu_5.4_O@CNDs, the carboxyl groups on the surface of CNDs were first converted to amides, and the amino groups were exposed on the surface of CNDs. Then, the aminated CNDs were reacted with PS, in which the amino and hydroxyl groups of the resulting CNDs (CNDs-B) were passivated **(Figure S3A)**. To investigate the contribution of carbonyl groups on the surface of CNDs to the formation of Cu_5.4_O@CNDs, sodium borohydride (NaBH_4_) was used to reduce the carbonyl groups to form hydroxyl groups on the surface of CNDs. The obtained Re-CNDs were reacted with PS followed by a hydrolysis process in 0.5 M sodium hydroxide solution. As a result, when the carbonyl group of CNDs was converted to hydroxyl groups, the hydroxyl group on the surface of Re-CNDs was converted to ether again, which led to the passivation of the carbonyl groups 25, 27. Next, Cu_5.4_O@CNDs were synthesized by replacing CNDs in synthesis with optimal doses of CNDs-A, CNDs-B, and Re-CNDs-A. The lyophilized Cu_5.4_O@CNDs-A and Cu_5.4_O@Re-CNDs-A were similar in character and were a dense black powder, in contrast to Cu_5.4_O@CNDs-B, which was sparse in texture and showed a cotton-like appearance **(Figure S3B)**.

Finally, we tested the radical scavenging catalytic activities of the synthesized Cu_5.4_O@CNDs-A, Cu_5.4_O@CNDs-B, and Cu_5.4_O@Re-CNDs-A, respectively, by the WST-1 kit. The results showed that the SOD enzyme activity of Cu_5.4_O@CNDs-A synthesized from CNDs-A with passivated hydroxyl groups was 1673 U/mg **(Figure S3C)**, The SOD enzyme activity of Cu_5.4_O@CNDs-B synthesized from CNDs-B with passivated hydroxyl and carboxyl groups was 2914 U/mg **(Figure S3D)**, The SOD enzyme activity of Cu_5.4_O@Re-CNDs-A synthesized from Re-CNDs-A after passivating the carbonyl group was 1635 U/mg **(Figure S3E)**. It was evidenced that passivation of the hydroxyl group alone makes the synthesis of Cu_5.4_O@CNDs blocked, and that the hydroxyl group has a positive contribution to the synthesis of Cu_5.4_O@CNDs; The enzyme activity of Cu_5.4_O@CNDs increased when the carboxyl group was simultaneously substituted, with partial recovery, suggesting that the presence of the carboxyl group forms a negative contribution to the synthesis of Cu_5.4_O@CNDs; The enzyme activity of Cu_5.4_O@CNDs decreased after passivation of the carbonyl group, suggesting a positive contribution of the carbonyl group to the formation of Cu_5.4_O@CNDs. These results indicate that carbonyl and hydroxyl groups promote Cu_5.4_O@CNDs, while the opposite is true for carboxyl groups. Therefore, we hypothesized that the hydroxyl and carboxyl groups on the surface of CNDs could form covalent coordination and ionic coordination bonds with Cu^2+^, which contributed to Cu^2+^ complexation on the surface of CNDs. When L-AA was added, the Cu^2+^ located on the surface of CNDs underwent a reduction reaction, and the Cu_5.4_O USNPs aggregated by Cu^0^ and Cu^1+^ were promoted to grow on the surface of CNDs by the adjustment of pH. We also believe that the SOD-like activity of CNDs is dependent on the carbonyl group coupled to the π-system and that electron transfer between the Cu_5.4_O USNPs and the carbonyl group of CNDs may occur during catalytic superoxide anion disproportionation. It was shown that the synergistic catalytic activity of Cu^0^ and Cu^1+^ arises from matching the rates of activation of the reactants, i.e., the rate of activation of H_2_ by Cu^0^ and the rate of activation of carbonyl-containing reactants by Cu^1+^. Where the carbonyl group is adsorbed at the center of Cu^1+^, the -CH_2_OH group attached to C=O will have strong electrostatic interactions with the oxygen vacancy center, causing the intermediate to form a downward adsorption conformation, elongating the C=O bond length and lowering the subsequent hydrogenation energy barrier 31.

In summary, Cu_5.4_O USNPs grow on the surface of CNDs through the combination of hydroxyl and carbonyl groups to generate Cu_5.4_O@CNDs. Cu_5.4_O USNPs make up for the deficiency of single enzyme activity of C-dots, which makes Cu_5.4_O@CNDs possess cascade nano-enzymatic activity. Moreover, compared with Cu_5.4_O USNPs and C-dots alone, the SOD-like enzyme activities of CNDs were greatly enhanced due to the synergistic activation of carbonyl-containing reactants by Cu^0^ and Cu^1+^, which lowered the hydrogenation barrier of the carbonyl group of the catalytic activity centers on the surface of CNDs. In addition, the binding of Cu^0^-Cu^1+^ to the carbonyl group increases its electron-hole separation, which makes the Cu_2_O coating on Cu NPs much more stable, which also contributes to the enhancement of ROS scavenging ability of Cu_5.4_O@CNDs 37.

### Cellular uptake and organ-targeted distribution capacity of Cu_5.4_O@CNDs

**Figure 4:**
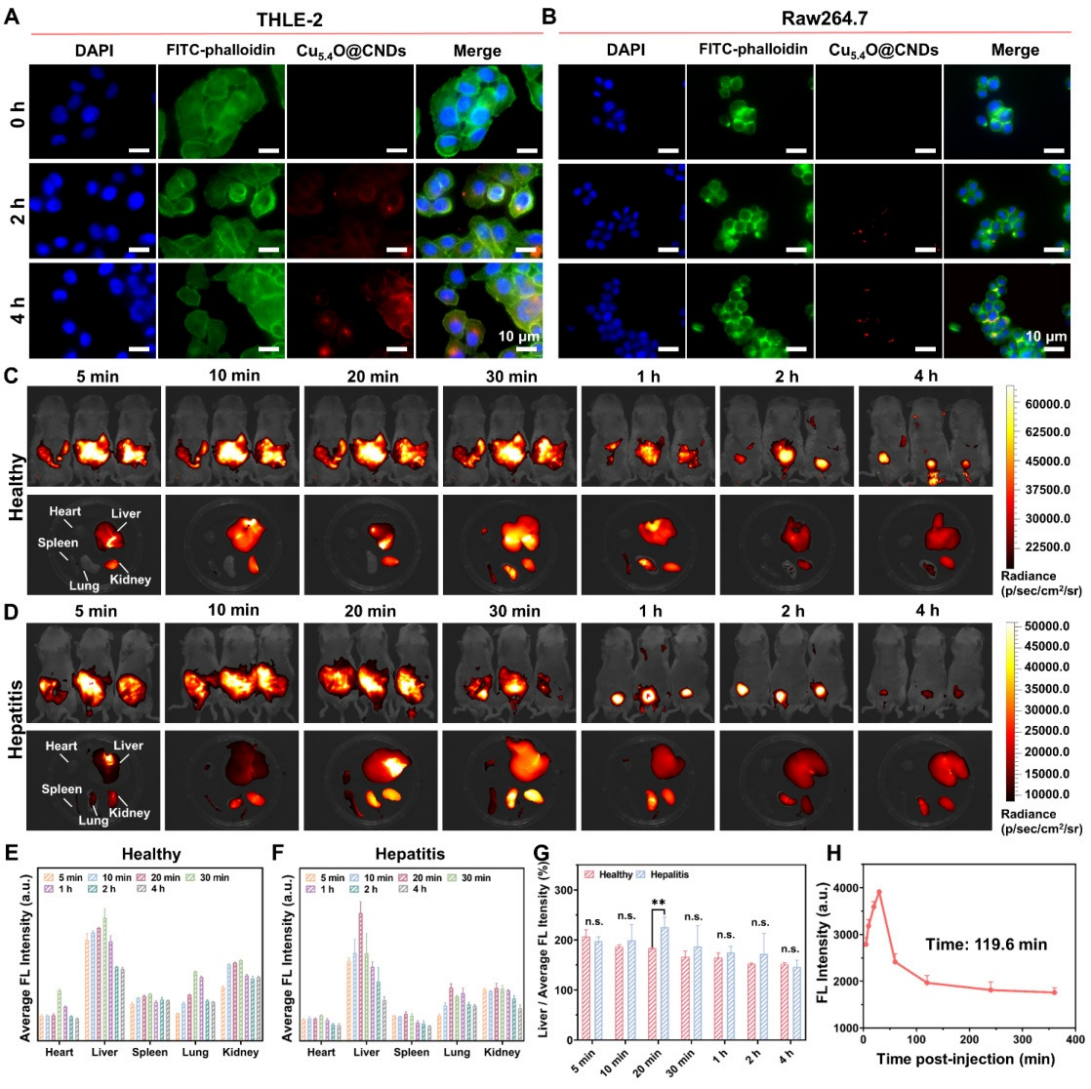
** Cellular uptake and biodistribution of Cu_5.4_O@CNDs.** (A) Fluorescence images of THLE-2 cells incubated with Cu_5.4_O@CNDs for 4 h. (B) Fluorescence images of Raw264.7 cells incubated with Cu_5.4_O@CNDs for 4 h. (C) Fluorescence imaging of healthy mice and organs at specified time points after intravenous injection of Cu_5.4_O@CNDs. (D) Fluorescence imaging of hepatitis mice and organs at specified time points after intravenous injection of Cu_5.4_O@CNDs. (E-F) Distribution of Cu_5.4_O@CNDs in major organs at different time points. (G) Difference of liver fluorescence level between healthy mice and hepatitis mice after intravenous injection of Cu_5.4_O@CNDs. (H) Average cellular fluorescence signals of blood at different time points after intravenous injection of Cu_5.4_O@CNDs. Data represent means ± s.d. from three independent replicates. ns: not significant, *P < 0.05, **P < 0.01, ***P < 0.001, one-way ANOVA.

To assess the cellular uptake of Cu_5.4_O@CNDs under increased ROS conditions, THLE-2 and Raw264.7 cells were treated with 100 μM H_2_O_2_. The results indicated a time-dependent uptake of the drug, reaching a maximum after 4 h of incubation** (Figure 4A-B)**.

The biodistribution and targeting capabilities of Cu_5.4_O@CNDs in healthy and damaged livers were further investigated *in vivo*. Cu_5.4_O@CNDs (0.5 mg/kg) were intravenously injected into both healthy and hepatitis mice, and their accumulation was monitored using fluorescence imaging at 0 to 4 h. A significant fluorescence signal was detected *in vivo* 5 min after injection and gradually diminished over the next 4 h. This indicated that Cu_5.4_O@CNDs circulated within the mice and were progressively eliminated from the body **(Figure 4C-D)**. Additionally, isolated organs were collected from the euthanized mice for further examination. Fluorescence imaging of isolated organs showed a rapid increase in Cu_5.4_O@CNDs accumulation in the liver 30 min post-injection, followed by a decline **(Figure 4C)**. In hepatitis model mice, Cu_5.4_O@CNDs rapidly accumulated in the liver 20 min after injection, followed by a decline **(Figure 4D)**, which demonstrated that Cu_5.4_O@CNDs could be uptaken by the damaged liver tissues more rapidly. Quantitative analysis of fluorescence data corroborated earlier findings, indicating that Cu_5.4_O@CNDs accumulation in the liver peaked 20 to 30 min post-injection. This suggests that administering Cu_5.4_O@CNDs prior to hepatic ischemia-reperfusion injury (HIRI) modeling offers an effective targeting strategy and a swift therapeutic window **(Figure 4E-F)**. Additionally, *ex vivo* fluorescence quantification of liver tissues showed that the fluorescence intensity of Cu_5.4_O@CNDs in damaged liver tissues was significantly higher than that in healthy mice, and the fluorescence intensity of the hepatitis model mice cleared faster than that of healthy mice** (Figure 4G)**. The above results demonstrated that Cu_5.4_O@CNDs contributes to its uptake and accumulation by the liver due to its properties of small size and negative charge. Importantly, owing to the proliferation of liver tissues and endothelial dysfunction of hepatic sinusoids in acute hepatitis, the uptake of Cu_5.4_O@CNDs by the liver in hepatitis was further promoted, which facilitated its entry into hepatocytes through hepatic sinusoids to play a role. Meanwhile, the increased GSH content in acute hepatitis liver tissues promoted GSH-mediated biotransformation, which led to an enhanced efficiency of Cu_5.4_O@CNDs clearance by the acute hepatitis liver **(Figure 4G)**. Finally, the pharmacokinetic study revealed that the blood fluorescence intensity gradually decreased following injection, with a half-life (t_1/2_) of 119.6 min, which also indicated that Cu_5.4_O@CNDs were readily excreted from the body within 24 h **(Figure 4H)**. In conclusion, Cu_5.4_O@CNDs exhibited excellent targeting and rapid accumulation in the liver.

### Anti-oxidative stress properties of Cu_5.4_O@CNDs *in vitro*

**Figure 5:**
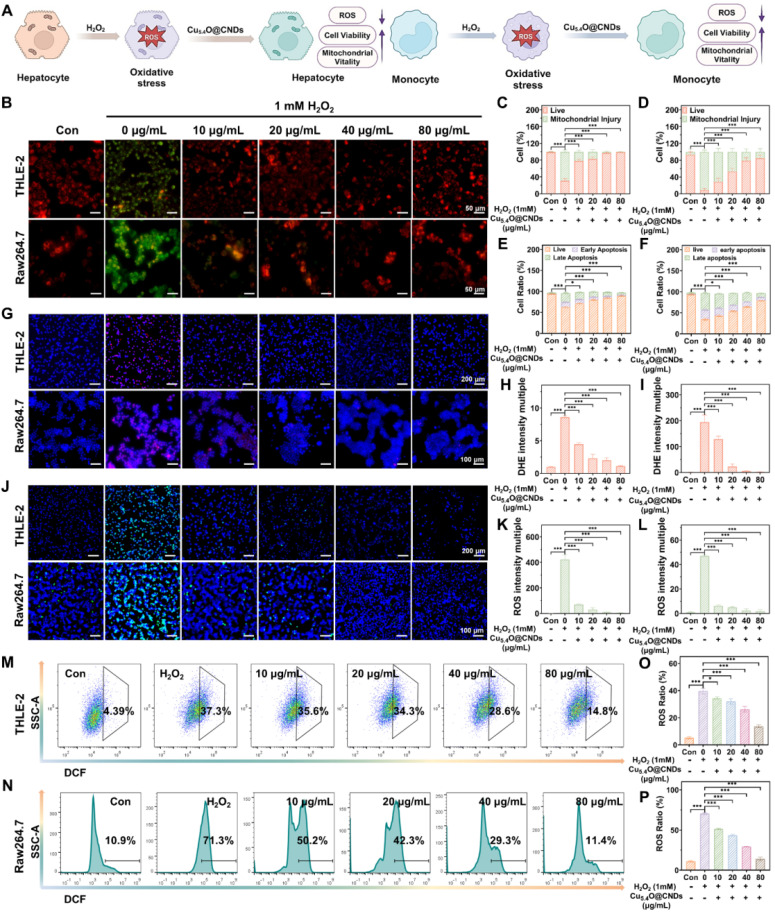
** Anti-oxidative stress properties of Cu_5.4_O@CNDs *in vitro*.** (A) Schematic representation of Cu_5.4_O@CNDs treatment of Oxidative stress cells. The figure was created partially with BioRender.com. (B) Representative JC-1 staining of THLE-2 and Raw264.7 cells under different treatment conditions. (C) Quantitative analysis of JC-1 fluorescent staining of THLE-2 cells. (D) Quantitative analysis of JC-1 fluorescent staining of Raw264.7 cells. (E) The quantitative flow cytometry results show cell apoptosis and necrosis distribution of THLE-2 cells under different treatment conditions. (F) The quantitative flow cytometry results show cell apoptosis and necrosis distribution of Raw264.7 cells under different treatment conditions. (G) Representative DHE staining of THLE-2 and Raw264.7 cells under different treatment conditions. (H) Quantitative analysis of DHE fluorescent staining of THLE-2 cells. (I) Quantitative analysis of DHE fluorescent staining of Raw264.7 cells. (J) Representative DCFH-DA staining of THLE-2 and Raw264.7 cells under different treatment conditions. (K) Quantitative analysis of DCFH-DA fluorescent staining of THLE-2 cells. (L) Quantitative analysis of DCFH-DA fluorescent staining of Raw264.7 cells. (M) The results by flow cytometry to ROS levels of THLE-2 cells under the indicated treatment conditions. (N) The results by flow cytometry to ROS levels of Raw264.7 cells under the indicated treatment conditions. (O) Quantitative analysis of flow cytometry to ROS levels of THLE-2 cells under the indicated treatment conditions. (P) Quantitative analysis of flow cytometry to ROS levels of Raw264.7 cells under the indicated treatment conditions. Data represent means ± s.d. from three independent replicates. ns: not significant, *P < 0.05, **P < 0.01, ***P < 0.001, one-way ANOVA.

Hepatic ischemia-reperfusion injury and acute liver injury not only lead to oxidative stress in hepatocytes but also can activate macrophages that have long been present in liver tissue 38. Hydrogen peroxide was utilized as an exogenous stimulus to quickly induce large amounts of ROS in THLE-2 and Raw264.7 cells **(Figure 5A)**. First, Cu_5.4_O@CNDs were evaluated for their protective capacity against oxidative stress cells. The JC-1 staining reagent accumulates in mitochondria based on membrane potential, displaying green fluorescence as monomers in early apoptotic cells and shifting to red fluorescence as J-aggregates in healthy cells 39. A decreased red/green fluorescence intensity ratio indicates early apoptosis. JC-1 staining indicated red fluorescence in both the blank THLE-2 and Raw264.7 cell groups. However, upon H_2_O_2_-induced oxidative stress, a significant increase in green fluorescence was observed, confirming ROS generation, mitochondrial membrane damage, and early apoptosis in the modeled cells **(Figure 5B)**. In the group pre-incubated with Cu_5.4_O@CNDs (0 ~ 80 μg/mL), intracellular green fluorescence signals significantly decreased as the concentration of Cu_5.4_O@CNDs increased **(Figure 5B)**. Quantitative fluorescence staining results confirmed that Cu_5.4_O@CNDs protect cells and mitigate oxidative stress-induced damage **(Figure 5C -D)**. Following H_2_O_2_ exposure, apoptotic cells constituted approximately 68.73 ± 4.45% in THLE-2 cells, whereas pretreatment with 80 μg/mL Cu_5.4_O@CNDs reduced this percentage to 0.58 ± 0.24% **(Figure 5C)**. The apoptotic cells accounted for about 91.4 ± 4.26% after H_2_O_2_ exposure in Raw.264.7 cells and 14.90 ± 7.30% in the 80 μg/mL Cu_5.4_O@CNDs pretreatment group** (Figure 5D)**.

Subsequently, apoptosis experiments were performed to determine the preventive effect of Cu_5.4_O@CNDs on apoptosis. Similar to the results of JC-1, the live cells accounted for about 62.67 ± 1.52% after H_2_O_2_ exposure in THLE-2 cells, while the percentage of apoptotic cells increased to 89.30 ± 1.11% in the 80 μg/mL Cu_5.4_O@CNDs pretreatment group **(Figure 5E)**. The live cells accounted for about 35.00 ± 2.39% after H_2_O_2_ exposure in Raw.264.7 cells, and 79.07 ± 0.35% in the 80 μg/mL Cu_5.4_O@CNDs pretreatment group **(Figure 5F)**.

Dihydroethidium (DHE), a red fluorescent probe specific for O_2_^•-^, was employed to evaluate the O_2_^•-^ scavenging capability of Cu_5.4_O@CNDs 40. DHE staining revealed no red fluorescence in the control group for both THLE-2 and Raw264.7 cells. In contrast, the H_2_O_2_-treated group showed prominent red fluorescence, which was notably diminished in the Cu_5.4_O@CNDs group in a concentration-dependent manner **(Figure 5G)**. Fluorescence staining analysis revealed that H_2_O_2_ exposure increased DHE production by 8.61 ± 0.20-fold in THLE-2 cells and 194.23 ± 27.88-fold in Raw264.7 cells compared to the control group. Following treatment with Cu_5.4_O@CNDs at 80 μg/mL, DHE levels in THLE-2 and Raw264.7 cells were reduced to 1.10 ± 0.08 and 2.23 ± 0.93 times the control levels, respectively **(Figure 5H-I)**.

Intracellular ROS levels were assessed using the oxidation-sensitive fluorescent dye 2′,7′-dichlorofluorescein (DCFH-DA). DCFH-DA is a non-fluorescent compound that permeates cell membranes and hydrolyzes into DCFH. Intracellular ROS oxidizes non-fluorescent DCFH to generate fluorescent DCF. Thus, the fluorescence of DCF intensity reflects the amount of intracellular ROS 41. The DCFH-DA staining showed that the blank cell group exhibited little green fluorescence. In contrast, a very bright green fluorescence appeared in the hydrogen peroxide-induced group, indicating that a large amount of ROS was successfully induced in the cells **(Figure 5J)**. Pre-incubation with Cu_5.4_O@CNDs (0-80 μg/mL) for 6 h significantly decreased intracellular green fluorescence intensity in a concentration-dependent manner, suggesting a substantial reduction in intracellular ROS content **(Figure 5J)**. Fluorescence staining analysis revealed that H_2_O_2_ exposure increased ROS production by 421.68 ± 38.19 times in THLE-2 cells and 19.41 ± 0.32 times in Raw264.7 cells compared to controls. Treatment with 80 μg/mL Cu_5.4_O@CNDs reduced ROS levels to 3.83 ± 0.88 times in THLE-2 cells and 1.78 ± 1.28 times in Raw264.7 cells relative to controls **(Figure 5K-L)**. Flow cytometry analysis indicated a concentration-dependent reduction in intracellular ROS levels in cells treated with varying concentrations of Cu_5.4_O@CNDs (0-80 μg/mL) compared to H_2_O_2_-stimulated cells **(Figure 5M-N)**. Quantitative statistics of the flow cytometry results suggested that 39.63 ± 2.14% of the THLE-2 cells induced by H_2_O_2_ cells generated ROS, and Raw264.7 cells induced 70.53 ± 1.00% **(Figure 5O-P)**. Following treatment with 80 μg/mL Cu_5.4_O@CNDs, ROS ratios were reduced to 13.67 ± 1.06% in THLE-2 cells and 14.07 ± 2.33% in Raw264.7 cells. It also indicated that Cu_5.4_O@CNDs had a significant concentration-dependent ROS scavenging effect **(Figure 5O-P)**.

### Anti-inflammatory properties of Cu_5.4_O@CNDs *in vitro*

**Figure 6:**
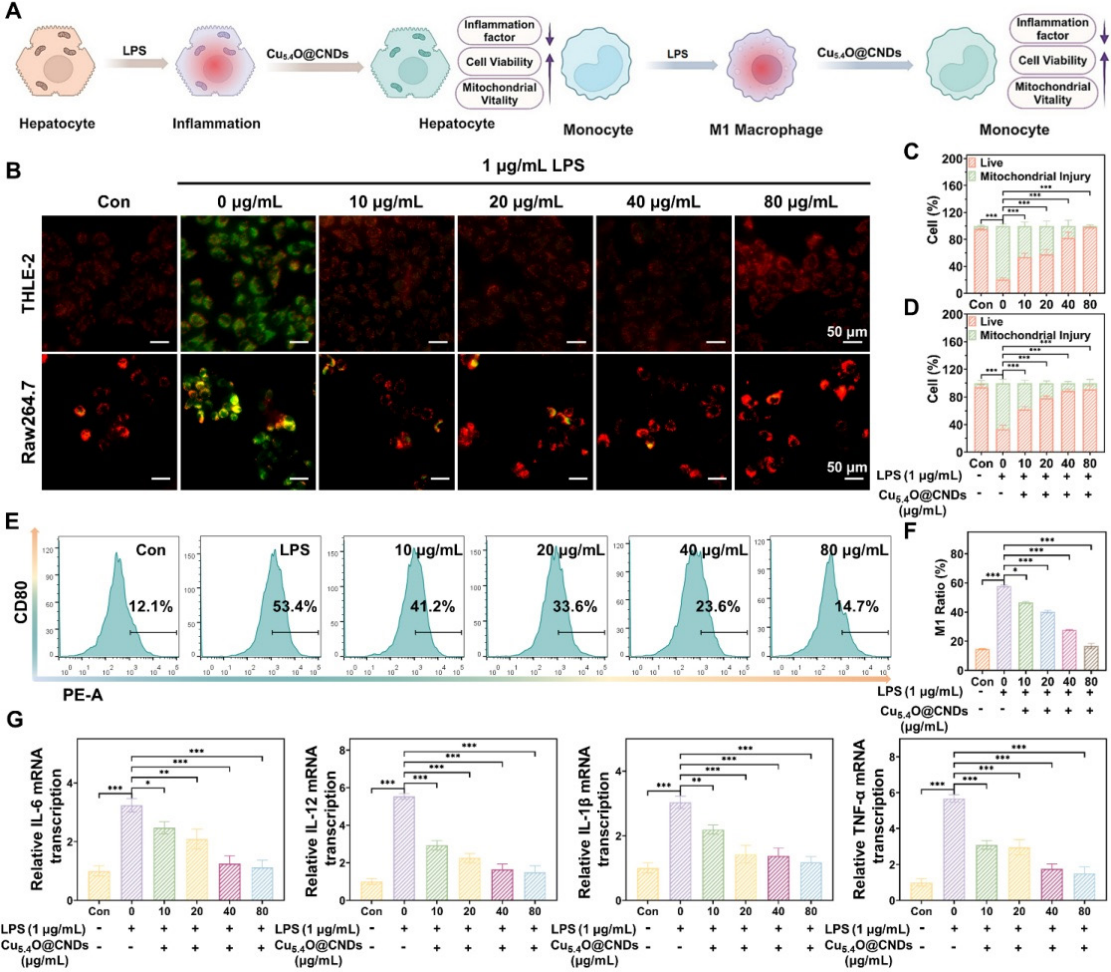
** Anti-inflammatory properties of Cu_5.4_O@CNDs* in vitro*.** (A) Schematic representation of Cu_5.4_O@CNDs treatment of LPS-induced inflammatory cells. The figure was created partially with BioRender.com. (B) Representative JC-1 staining of THLE-2 & Raw264.7 cells under different treatment conditions. (C) Quantitative analysis of JC-1 fluorescent staining of THLE-2 cells. (D) Quantitative analysis of JC-1 fluorescent staining of Raw264.7 cells. (E) The results by flow cytometry to CD80 levels of Raw264.7 cells under different treatment conditions. (F) Quantitative analysis of flow cytometry to CD80 levels of Raw264.7 cells. (G) Relative expression of mRNAs for inflammatory cytokines of IL-1β, IL-6, IL-12, and TNF-α. Data represent means ± s.d. from three independent replicates. ns: not significant, *P < 0.05, **P < 0.01, ***P < 0.001, one-way ANOVA.

Inflammatory responses often occur during oxidative stress, and lipopolysaccharide (LPS) activates macrophages through cell signaling systems, inducing a variety of cytokines and inflammatory mediators, which leads to an inflammatory state in cells 42 **(Figure 6A)**. The protective effect of Cu_5.4_O@CNDs on inflammatory cells was assessed using JC-1 in both THLE-2 and Raw264.7 cell lines. In the control group, cells exhibited red fluorescence. However, upon LPS-induced inflammation, significant green fluorescence was observed, indicating mitochondrial membrane damage and early apoptosis in the modeled cells** (Figure 6B)**. After pre-incubation with Cu_5.4_O@CNDs, the intensity of green fluorescence was reduced, proving the improvement of cell status **(Figure 6B)**. Quantitative analysis revealed that in THLE-2 cells, the apoptotic cell ratio was 79.83 ± 2.56% in the LPS group, which significantly decreased to 1.98 ± 1.76% with 80 μg/mL Cu_5.4_O@CNDs pretreatment **(Figure 6C)**. The apoptotic cells ratio was 66.77 ± 5.57% after LPS exposure in Raw.264.7 cells, and 8.93 ± 5.49% in the 80 μg/mL Cu_5.4_O@CNDs pretreatment group **(Figure 6D)**.

Macrophage activation and polarization into various subpopulations are influenced by distinct microenvironments, with LPS induction typically transforming macrophages into M1-type, which enhances inflammation 43. The impact of Cu_5.4_O@CNDs on macrophage polarization was confirmed by examining cytomorphological changes in M1 macrophages, indicated by the overexpression of surface CD80. Flow cytometry analysis indicated that Cu_5.4_O@CNDs suppressed the conversion of pro-inflammatory M1 macrophages, leading to decreased expression of inflammatory mediators and a reduced cellular inflammatory response **(Figure 6E)**. Quantitative statistics showed that LPS cells induced 57.80 ± 0.70% conversion of Raw264.7 cells into M1 macrophages **(Figure 6F)**. Real-time quantitative polymerase chain reaction (qPCR) was employed to measure the relative transcription levels of TNFα, IL-6, IL-12, and IL-1β. The study demonstrated a significant increase in cellular inflammatory factors following LPS induction, which was notably reduced after pre-incubation with Cu_5.4_O@CNDs **(Figure 6G)**.

### Biocompatibility evaluation of Cu_5.4_O@CNDs

**Figure 7:**
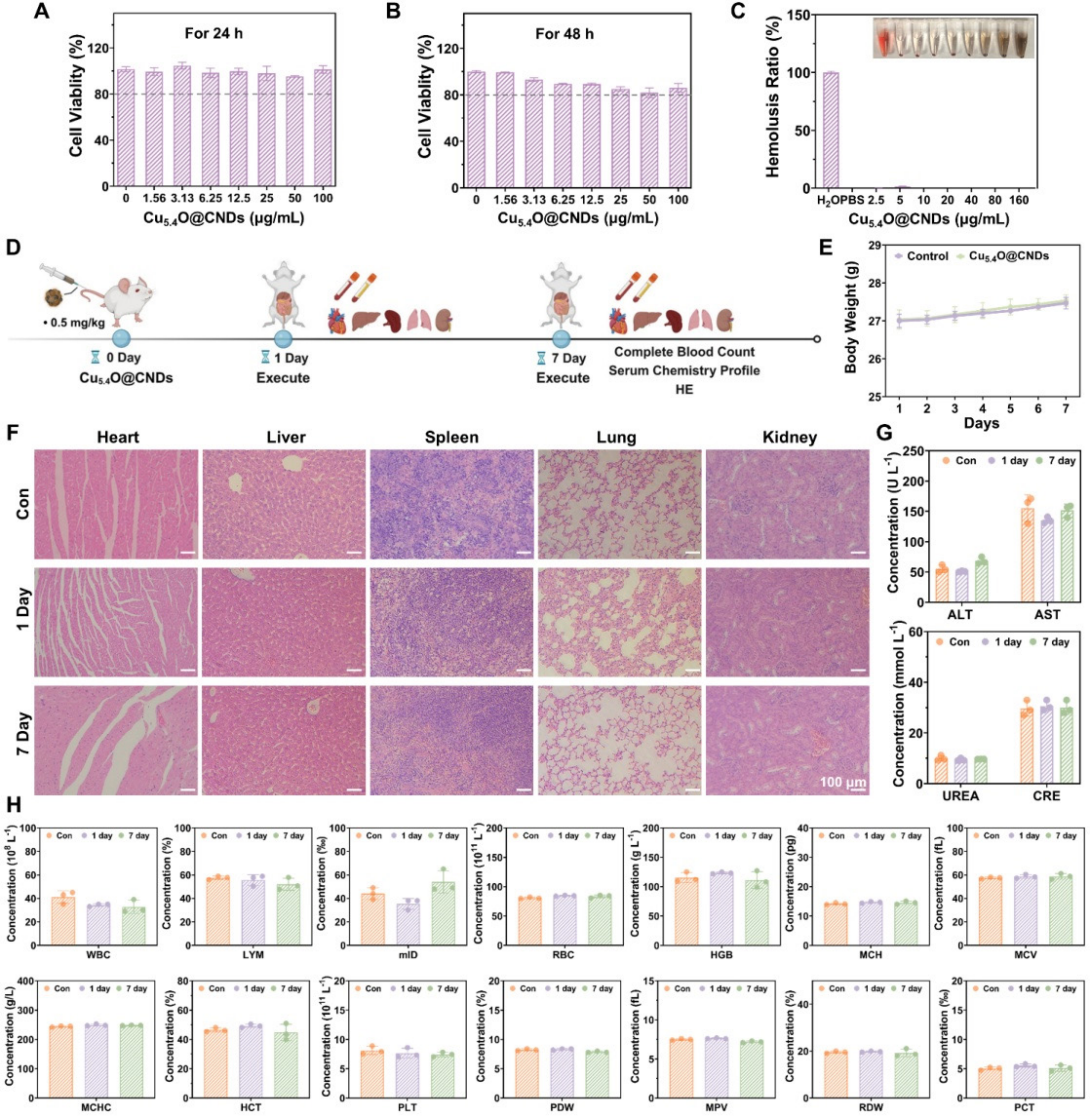
** Biocompatibility of Cu_5.4_O@CNDs.** (A) THLE-2 cell viability after incubation for 24 h with Cu_5.4_O@CNDs. (B) THLE-2 cell viability after incubation for 48 h with Cu_5.4_O@CNDs. (C) The ratio of hemolysis in the subgroups. (D) Schematic diagram of biocompatibility experiment. The figure was created partially with BioRender.com. (E) Weight variation of normal mice at 7 days after treatment with Cu_5.4_O@CNDs. (F) Evaluation of *in vivo* toxicity of Cu_5.4_O@CNDs to major organs (heart, liver, spleen, lung, and spleen) at 1 day and 7 days after intravenous administration. (G) Serum levels of liver function indicators: alanine transaminase (ALT) and aspartate transaminase (AST). Serum levels of kidney function indicators: blood urea nitrogen (UREA) and creatinine (CRE). (H) Blood parameters in normal mice (control group) and mice intravenously injected with Cu_5.4_O@CNDs. Data represent means ± s.d. from three independent replicates. ns: not significant, *P < 0.05, **P < 0.01, ***P < 0.001, one-way ANOVA.

Since biocompatibility is important for the potential clinical applications of nanomaterials, the toxicity of Cu_5.4_O@CNDs was evaluated *in vitro*. Cell viability in THLE-2 cells exposed to various concentrations of Cu_5.4_O@CNDs for 24 h was assessed using the MTT assay, revealing nearly 100% viability even at 100 μg/mL **(Figure 7A)**. When the incubation time reached 48 h, the cell viability was still higher than 80%, which proved the good safety of Cu_5.4_@CNDs *in vitro* **(Figure 7B)**. Next, the hemolysis assay was used to detect the biocompatibility of Cu_5.4_O@CNDs, and the results showed that upon the concentration was 160 μg/mL, the hemolysis rate was still below 5%, which suggested that the nanomaterials didn't cause hemolysis of blood cells and were safe for intravenous injection **(Figure 7C)**.

In addition, 0.5 mg/kg was used as the concentration for all *in vivo* biocompatibility evaluation experiments. The *in vivo* biocompatibility of Cu_5.4_O@CNDs was assessed in healthy mice by examining blood chemistry and major organ histopathology **(Figure 7D)**. Following a 7-day regimen of Cu_5.4_O@CNDs injections, samples from major organs (heart, liver, spleen, lungs, and kidneys), serum, and plasma were collected on days 1 and 7, with body weight monitored throughout the study. The body weight record results exhibited that Cu_5.4_O@CNDs did not affect the status and body weight of the mice** (Figure 7E)**. H&E staining results showed no obvious signs of damage in the tissues, suggesting that Cu_5.4_O@CNDs had good biocompatibility **(Figure 7F)**. Serum biochemical analysis showed that liver function indicators (AST and ALT) and kidney function indicators (UREA and CRE) in the Cu_5.4_O@CNDs injected group were similar to the control group, indicating liver and kidney biocompatibility **(Figure 7G)**. Whole blood cell analysis in mice revealed no statistically significant differences from the control group **(Figure 7H)**. The studies uniformly demonstrated that synthesized Cu_5.4_O@CNDs exhibited minimal *in vivo* toxicity both in the short and long term.

### *In vivo* therapeutic efficacy of Cu_5.4_O@CNDs on HIRI mice

**Figure 8:**
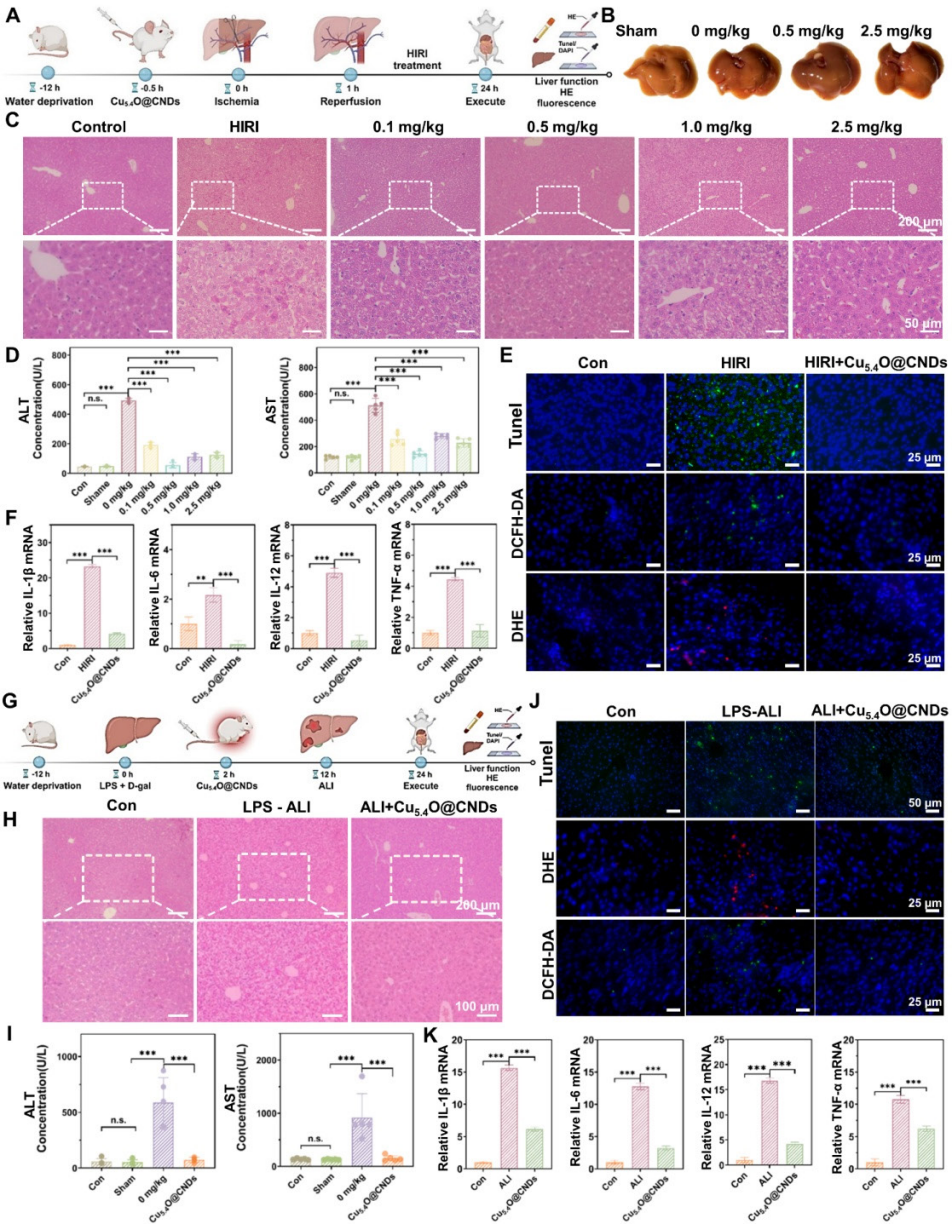
***In vivo* therapeutic efficacy of Cu_5.4_O@CNDs on HIRI & LPS-ALI mice.** (A) Schematic illustration of the establishment and treatment schedule of HIRI mice. The figure was created partially with BioRender.com. (B) Optical image of liver tissue. (C) H&E staining (Scale bar: 100 μm & 50 μm) of liver tissues. (D) Serum levels of ALT and AST in HIRI mice at 24 h after different treatments. (E) TUNEL assay, DCFH-DA, and DHE staining (Scale bar: 25 μm) of liver tissues. (F) Relative expression of mRNAs for cytokines of IL-1β, IL-6, IL-12, TNF-α. (G) Schematic illustration of the establishment and treatment schedule of LPS-ALI mice. The figure was created partially with BioRender.com. (H) H&E staining (Scale bar: 200 μm & 100 μm) of liver tissues. (I) Serum levels of ALT and AST in LPS-ALI mice at 12 h after different treatments. (J) TUNEL assay, DCFH-DA, and DHE staining (Scale bar: 25 μm) of liver tissues. (K) Relative expression of mRNAs for cytokines of IL-1β, IL-6, IL-12, TNF-α. Data represent means ± s.d. from three independent replicates. ns: not significant, *P < 0.05, **P < 0.01, ***P < 0.001, one-way ANOVA.

Based on the anti-oxidative stress and anti-inflammatory effects of Cu_5.4_O@CNDs *in vitro*, the protective effects of Cu_5.4_O@CNDs in the mouse model of HIRI were further investigated. According to the results analyzed above and the current consensus, excessive oxidative stress and inflammation contribute to the pathology of HIRI 34. Firstly, the HIRI mice model was established **(Figure 8A)**. Then, the optimal treatment dose of Cu_5.4_O@CNDs was determined. Liver tissues were photographed, showing that some tissue damage surfaces were visible, and the liver status of the 0.5 mg/kg pretreatment group was better than that of the disease modeling group** (Figure 8B)**. Meanwhile, the H&E staining images of mouse liver tissues showed that the HIRI modeling group's cells appeared to punctate hepatocyte necrosis, some intracapillary cholestasis, and inflammatory cell infiltration** (Figure 8C)**. Hepatic histopathological manifestations were significantly improved in the pre-injected Cu_5.4_O@CNDs group, and minimal areas of hepatocyte necrosis and cytolysis were observed in the HIRI mouse model treated with 0.5 mg/kg of Cu_5.4_O@CNDs **(Figure 8C)**. Liver function tests in mice revealed elevated alanine transaminase (ALT) and aspartate aminotransferase (AST) levels in the HIRI model group compared to controls. However, pre-injection with Cu_5.4_O@CNDs significantly reduced ALT and AST levels, indicating a protective effect on liver function **(Figure 8D)**. At a concentration of 0.5 mg/kg, Cu_5.4_O@CNDs resulted in liver function indices in mice that closely matched those of the control group, suggesting this as the optimal therapeutic dose **(Figure 8D)**. In summary, the Cu_5.4_O@CNDs demonstrated a protective effect on hepatic function in the HIRI mice model, with 0.5 mg/kg identified as the optimal therapeutic concentration for further studies.

TUNEL staining of frozen tissue sections was used to evaluate hepatocyte necrosis and apoptosis. The results showed a significant increase in green fluorescence in the HIRI group, while no notable difference was observed between the pretreated and control groups, suggesting that Cu_5.4_O@CNDs effectively protect hepatocytes and hepatic function **(Figure 8E)**. The *in vivo* ROS removal capability of Cu_5.4_O@CNDs was assessed using the ROS-specific fluorescent dyes DCFH-DA and DHE for imaging. The green fluorescence of DCFH-DA was significantly increased in liver tissues after hepatic ischemia-reperfusion, and the ROS level was reduced in the tissues stained by Cu_5.4_O@CNDs pre-injection** (Figure 8E)**. Meanwhile, DHE was also seen to increase the intensity of red fluorescence in the modeling group and recover the intensity of staining fluorescence in the pretreatment group **(Figure 8E)**. The results indicated that Cu_5.4_O@CNDs could alleviate liver function injury and hepatocyte apoptosis by removing ROS in liver ischemia-reperfusion injury disease.

In addition, mouse liver tissues were processed to extract the relevant RNA to verify the changes in the transcription levels of common pro-inflammatory cytokines. After successful modeling, levels of the inflammatory factors IL-1β, IL-6, IL-12, and TNF-α significantly increased. However, in the group pre-injected with Cu_5.4_O@CNDs, inflammation levels remained comparable to those in healthy control mice **(Figure 8F)**. The findings demonstrated that Cu_5.4_O@CNDs effectively mitigated the inflammatory response by scavenging ROS, thereby preventing oxidative stress and inflammation in hepatic ischemia-reperfusion injury.

### *In vivo* therapeutic efficacy of Cu_5.4_O@CNDs on LPS-ALI mice

To verify that Cu_5.4_O@CNDs can be applied to other acute liver injury diseases, an LPS-induced acute liver injury model (LPS-ALI) was established. Cu_5.4_O@CNDs (0.5 mg/kg) was administered 6 h post LPS + D-gal induction, followed by euthanasia 12 h later to collect liver tissues and serum for liver function analysis **(Figure 8G)**. Similar to the HIRI model, the H&E staining images of mouse liver tissues showed that in the LPS-induced hepatitis model, the hepatocytes showed apparent balloon-like lesions accompanied by a large number of inflammatory cell infiltrations. The hepatic histopathological manifestations of the Cu_5.4_O@CNDs-treated group were markedly improved, and the inflammatory symptoms were reduced **(Figure 8H)**. Biochemical tests on mouse serum revealed elevated liver function indexes in LPS-induced hepatitis mice, indicating severe liver injury. In contrast, the Cu_5.4_O@CNDs treatment group showed reduced liver function indexes, approaching those of the control group, demonstrating a significant therapeutic effect **(Figure 8I)**.

TUNEL staining of frozen tissue sections was conducted to evaluate hepatocyte necrosis and apoptosis. The modeling group exhibited significantly increased TUNEL green fluorescence, while no notable difference was observed between the pretreatment and control groups, indicating that Cu_5.4_O@CNDs possess a strong anti-inflammatory effect **(Figure 8J)**. To evaluate the ROS scavenging ability in the ALI-affected liver of Cu_5.4_O@CNDs, frozen sections were stained with DCFH-DA and DHE. LPS-induced hepatitis showed a significant increase in DCFH-DA green fluorescence, while Cu_5.4_O@CNDs treatment reduced ROS levels **(Figure 8J)**. Meanwhile, high intensity of red fluorescence in hepatitis tissues was seen by DHE, and the fluorescence intensity of the tissues decreased after treatment. The results suggested that Cu_5.4_O@CNDs can achieve relief of inflammation by scavenging ROS in LPS-induced acute hepatitis disease **(Figure 8J)**.

Mouse liver tissues were processed to extract RNA for verifying changes in common pro-inflammatory cytokine expression levels. The study found that four common cytokines were significantly elevated following successful modeling, while inflammation levels in the Cu_5.4_O@CNDs-injected group were comparable to those in healthy control mice **(Figure 8K)**. The study indicates that Cu_5.4_O@CNDs can mitigate oxidative stress and inflammation in acute hepatitis by eliminating ROS *in vivo*.

### Therapeutic mechanisms of Cu_5.4_O@CNDs on HIRI & LPS-ALI

To further elucidate the potential therapeutic mechanism, after the mice were executed and the frozen liver tissues were obtained, RNA extraction and purification, RNA fragmentation and reverse transcription, construction of cDNA libraries, high-throughput sequencing and other experimental procedures were carried out, and the results were subjected to gene and protein differential expression analyses, which were able to reveal the comprehensive gene expression information of the liver tissues of all the groups in the Cu_5.4_O@CNDs intervention model, and analyze the changes of gene expression under different conditions. It can reveal the comprehensive gene expression information of each group in the Cu_5.4_O@CNDs intervention model, analyze the expression changes of differentially expressed genes under different conditions, and screen the differentially expressed genes and corresponding signaling pathways in the HIRI group and the Cu_5.4_O@CNDs prevention group, and the LPS-ALI group and the Cu_5.4_O@CNDs treatment group, respectively.

**Figure 9:**
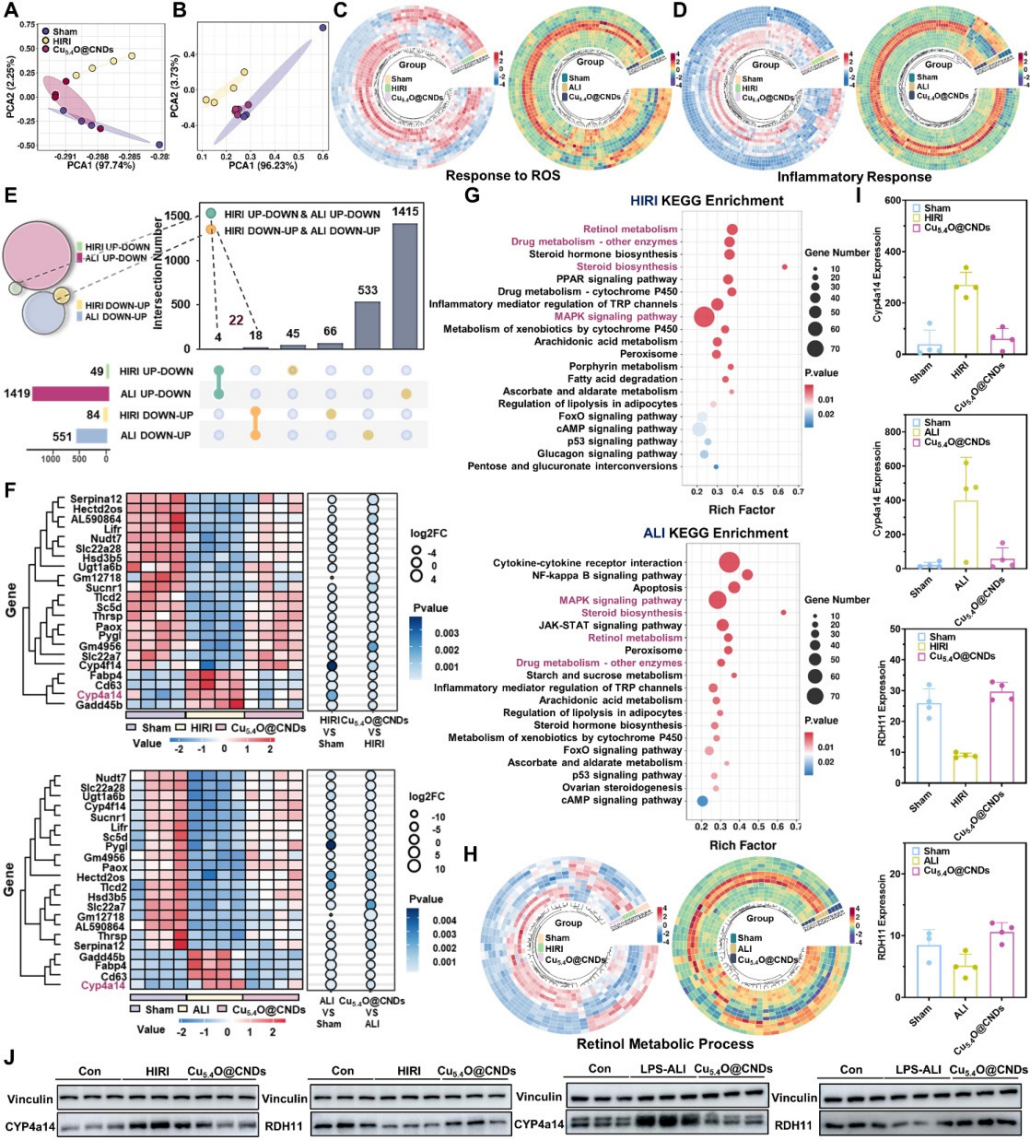
** Therapeutic mechanisms of Cu_5.4_O@CNDs on HIRI & LPS-ALI.** (A) PCoA of the DEGs between the Sham group, HIRI group, and Cu_5.4_O@CNDs group (n = 4), each point represented one mouse. (B) PCoA of the DEGs between the Sham group, LPS-ALI group, and Cu_5.4_O@CNDs group (n = 4), each point represented one mouse. (C) Differential gene heat maps associated with Response to ROS (fold change≥2 and P< 0.01). (D) Differential gene heat maps related to inflammatory response (fold change≥2 and P< 0.01). (E) Differential genes with the same trend in HIRI and LPS-ALI. (F) Differential gene heat maps with the same trend in HIRI and LPS-ALI. (G) KEGG pathway enrichment analysis. The 20 most significantly enriched pathways were shown. (H) Differential gene heat maps associated with Retinol metabolic process (fold change≥2 and P< 0.01). (I) Expression difference of CYP4a14 and RDH11 genes in HIRI and LPS-ALI diseases, respectively. (J) Liver protein expression of CYP4a14 and RDH11 (n = 3).

Firstly, the graphs were made by transcriptomics PCA analysis which showed that there was a high reproducibility between samples within the experimental groups, the healthy mice in the control group had a significant difference and low correlation with the mice in the modeling group, and the mice in the treatment or prevention group had a high correlation with the healthy group **(Figure 9A-B)**. It proved that HIRI and LPS-ALI modeling was successful. Differential gene expression (DGE) analysis remains an essential application of RNA-seq as a traditional research tool. The Wayne plots suggested that Cu_5.4_O@CNDs were associated with a total of 166 genes related to the treatment of HIRI disease **(Figure S4A)**, and Cu_5.4_O@CNDs were associated with a total of 1978 genes related to the treatment of LPS-ALI disease **(Figure S5A)**. The volcano map analysis revealed that in the Cu_5.4_O@CNDs and HIRI group, there were 900 up-regulated and 450 down-regulated genes, while in the Cu_5.4_O@CNDs and LPS-ALI group, there were 1,421 up-regulated and 2,719 down-regulated genes **(Figures S4B and S5B)**. Gene clustering and analysis revealed differential expression levels between the two disease models, as well as variations in the expression of the same genes across control, model, and treatment groups** (Figures S4C and S5C)**. GO and KEGG analyses were conducted following the sequencing results. First, according to GO analysis, significant differences in metabolic processes related to redox, immune system, inflammatory response, and defense against viruses were found between the HIRI disease and treatment models **(Figure S4D-E)**, and significant differences in metabolic processes related to redox, immune system, and cholesterol were found between the LPS-ALI disease and treatment models **(Figure S5D-E)**. The findings indicate that Cu_5.4_O@CNDs could be involved in modulating hepatic oxidative stress and inflammatory pathways, potentially aiding in the prevention and treatment of liver diseases. KEGG analysis identified enriched pathways among differential genes in the control-disease-treatment groups, including PI3K-Akt, MAPK, Rap1 signaling, and retinol metabolism pathways, which are classical inflammatory and apoptotic pathways **(Figures S4F and S5F)**. And Bubble plots suggested that treating acute liver injury disease by Cu_5.4_O@CNDs may be associated with multiple inflammatory signaling pathways, and retinol metabolism pathways **(Figure S4G and S5G)**. The above evidence and results demonstrated that Cu_5.4_O@CNDs could regulate hepatitis inflammation and oxidative stress processes in the prevention and treatment of liver diseases.

The above RNA sequencing analysis of the modeling group and healthy mice demonstrated that ROS metabolic processes, inflammatory response pathways, and retinol metabolism are highly correlated in the pathogenesis of two acute liver injury diseases, HIRI and LPS-ALI. Accordingly, Cu_5.4_O@CNDs were designed to target their pathogenesis. To assess the therapeutic efficacy, differential gene heat map clustering analysis was conducted on ROS and inflammation response-related genes. The analysis revealed distinct gene expression patterns among the control, model, and treatment groups, demonstrating that Cu_5.4_O@CNDs effectively modulate genes influencing our target genes **(Figure 9C-D)**.

To further analyze the common therapeutic mechanism of Cu_5.4_O@CNDs in HIRI and LPS-ALI, the two RNA sequencing models were screened in the order of control-model-treatment to find out the differential genes that "increase-decrease" or "decrease-increase". The results indicated that both HIRI and LPS-ALI models exhibited four differential genes with an 'ascending-descending' pattern in the control-model-treatment group sequence. In the HIRI and LPS-ALI models, 18 genes showed a "decrease-increase" in the control-model-treatment group **(Figure 9E)**.

Therefore, the total number of differential genes with the same trend was 22. At the same time, these 22 genes were clustered and analyzed, which can visualize the expression level and enrichment degree of different genes among different groups **(Figure 9F)**. Subsequently, based on the screening results, KEGG analysis of the target genes was also performed, and the enrichment results showed that the lipid metabolism-related pathways such as "cholesterol metabolism, retinol metabolism", the classical inflammatory response pathways such as "MAPK signaling pathway", drug metabolism pathway, etc. were highly enriched, which proved that the expression level of different genes in different groups was highly enriched. The results showed that lipid metabolism-related pathways such as "cholesterol metabolism, retinol metabolism", classical inflammatory response pathways such as "MAPK signaling pathway", and drug metabolism pathways were highly enriched, which demonstrated that the therapeutic mechanism of Cu_5.4_O@CNDs was common to HIRI and LPS-ALI. Interestingly, the pathogenesis common to both acute liver injury diseases, HIRI and LPS-ALI, i.e., is highly associated with disorders of ROS metabolic processes, inflammatory response pathways, and retinol metabolism. The pathogenesis corresponds to the therapeutic mechanism, demonstrating that the stabilization of the retinol metabolic pathway may be a therapeutic pathway specific for Cu_5.4_O@CNDs, which showed that there is a difference in the expression of the same genes in the control-model-treatment group for the retinol metabolic pathway **(Figure 9G)**. Notably, there were 3 genes associated with the retinol metabolic pathway among the 22 differential genes, and the gene CYP4a14 was screened based on enrichment, differential level, and gene function **(Figure 9H)**. CYP4a14 is a retinoic acid degradation-associated gene 44. Up-regulation of CYP4a14 expression and down-regulation of rate-limiting enzymes of retinol metabolism, such as RDH11, and retinol dehydrogenase, resulted in retinol metabolism disorders characterized by tissue retinol overload and reduction of all-trans-retinol 45. This promoted metabolic disorders in liver tissues and tissue toxicity **(Figure 9I)**. Based on relative mRNA expression, we further explored the protein level of CYP4a14 and RDH11. The results demonstrated that both HIRI and LPS-ALI diseases were associated with up-regulation of the retinol metabolism gene CYP4a14 and its protein expression, accompanied by down-regulation of RDH11. The intervention of Cu_5.4_O@CNDs reversed retinol metabolism disorders, resulting in the restoration of the CYP4a14 and RDH11 genes and their protein expression **(Figure 9J and Figure S6).**

The results of the above RNA sequencing analyses and immunoblotting results demonstrated that Cu_5.4_O@CNDs could not only remove excessive ROS through its cascade nano-enzymatic activity but also prevent and alleviate ROS infiltration by regulating ROS-responsive pathway-related genes; at the same time, Cu_5.4_O@CNDs could ameliorate the metabolic disorders of retinol metabolism and tissue retinol overload through the down-regulation of CYP4a14; furthermore, Cu_5.4_O@CNDs could improve the metabolic disorders of retinol metabolism by down-regulation of CYP4a14; and Cu_5.4_O@CNDs simultaneously modulated multiple classical inflammatory pathways and attenuated the inflammation level in liver tissues and body forces. The above evidence and results demonstrate that Cu_5.4_O@CNDs can regulate hepatic retinol metabolism, hepatitis inflammation, and oxidative stress processes in the prevention and treatment of liver diseases.

## Conclusion

In summary, the synthesized Cu_5.4_O@CNDs nanozyme particles exhibit cascade-amplified enzyme activity, are cost-effective and straightforward to produce, and can treat various acute liver injury diseases. And it showed good biocompatibility. Cu_5.4_O@CNDs can be enriched in the mitochondrial membrane of damaged liver-targeted cells by scavenging oxidative stress products. Transcriptome sequencing was employed to explore the specific mechanisms of Cu_5.4_O@CNDs in treating acute liver injury diseases. This treatment involves down-regulating CYP4a14, correcting retinol metabolism disorders and overload, regulating ROS-responsive pathway genes, mitigating ROS infiltration, and modulating inflammatory responses. In conclusion, multiple acute liver injury disease models were established to explore specific regulatory pathways, provide new therapeutic targets for oxidative stress-related diseases, and construct a new type of nano-enzymatic material, providing a promising biomedical therapeutics strategy.

## Supplementary Material

Supplementary materials and methods, figures and tables.

## Abbreviations

TEM: transmission electron microscopy
FT-IR: Fourier transform infrared spectroscopy
O_2_^•-^: superoxide anion
·OH: Hydroxyl radicals
NBT: nitro blue tetrazolium
TMB: tetramethylbenzidine
ABTS^•+^: 2,2'-azino-bis (3-ethylbenzthiazoline-6-sulfonic acid)
GSEA: Gene Set Enrichment Analysis
ALF: acute liver failure
DCFH-DA: dichlorodihydrofluorescein diacetate
DHE: dihydroethidium
ALT: alanine aminotransferase
AST: aspartate aminotransferase
DEG: differentially expressed genes
LPS: lipopolysaccharide
D-GalN: D-galactosamine
TUNEL: terminal deoxynucleotidyl transferase-mediated deoxyuridine triphosphate nick end labeling
MMP: mitochondrial membrane potential
IL-1β: interleukin-1β
IL-6: interleukin-6
TNF-α: tumor necrosis factor-α
KEGG: the Kyoto Encyclopedia of Genes and Genomes
PCA: principal component analysis

## References

[1] Bernal W, Lee WM, Wendon J, Larsen FS, Williams R (2015). Acute liver failure: A curable disease by 2024?. J Hepatol.

[2] Wendon J, Cordoba J, Dhawan A, Larsen FS, Manns M, Samuel D (2017). Easl clinical practical guidelines on the management of acute (fulminant) liver failure. J Hepatol.

[3] Reuben A, Tillman H, Fontana RJ, Davern T, Mcguire B, Stravitz RT (2016). Outcomes in adults with acute liver failure between 1998 and 2013. Ann Intern Med.

[4] Bernal W, Wendon J (2013). Acute liver failure. N Engl J Med.

[5] Bajaj JS, Cordoba J, Mullen KD, Amodio P, Shawcross DL, Butterworth RF (2011). Review article: The design of clinical trials in hepatic encephalopathy - an international society for hepatic encephalopathy and nitrogen metabolism (ishen) consensus statement. Aliment Pharmacol Ther.

[6] Stravitz RT, Lee WM (2019). Acute liver failure. Lancet.

[7] Parola M, Pinzani M (2019). Liver fibrosis: Pathophysiology, pathogenetic targets and clinical issues. Mol Aspects Med.

[8] Hernansanz-Agustín P, Choya-Foces C, Carregal-Romero S, Ramos E, Oliva T, Villa-Piña T (2020). Na+ controls hypoxic signalling by the mitochondrial respiratory chain. Nature.

[9] Wang Z, Ying Z, Bosy-Westphal A, Zhang J, Schautz B, Later W (2010). Specific metabolic rates of major organs and tissues across adulthood: Evaluation by mechanistic model of resting energy expenditure. Am J Clin Nutr.

[10] Gracia-Sancho J, Caparrós E, Fernández-Iglesias A, Francés R (2021). Role of liver sinusoidal endothelial cells in liver diseases. Nat Rev Gastroenterol Hepatol.

[11] Yan M, Huo Y, Yin S, Hu H (2018). Mechanisms of acetaminophen-induced liver injury and its implications for therapeutic interventions. Redox Biol.

[12] Wang C, Tao Q, Wang X, Wang X, Zhang X (2016). Impact of high-fat diet on liver genes expression profiles in mice model of nonalcoholic fatty liver disease. Environ Toxicol Pharmacol.

[13] Ogawa Y, Kurosu H, Yamamoto M, Nandi A, Rosenblatt KP, Goetz R (2007). Βklotho is required for metabolic activity of fibroblast growth factor 21. Proc Natl Acad Sci U S A.

[14] Trivedi P, Wang S, Friedman SL (2021). The power of plasticity-metabolic regulation of hepatic stellate cells. Cell Metab.

[15] Romeo S, Valenti L (2016). Regulation of retinol-binding protein 4 and retinol metabolism in fatty liver disease. Hepatology.

[16] Isoherranen N, Zhong G (2019). Biochemical and physiological importance of the cyp26 retinoic acid hydroxylases. Pharmacol Ther.

[17] Mansouri A, Gattolliat CH, Asselah T (2018). Mitochondrial dysfunction and signaling in chronic liver diseases. Gastroenterology.

[18] Yu Z, Lou R, Pan W, Li N, Tang B (2020). Nanoenzymes in disease diagnosis and therapy. Chem Commun (Camb).

[19] Poilil Surendran S, George Thomas R, Moon MJ, Jeong YY (2017). Nanoparticles for the treatment of liver fibrosis. Int J Nanomedicine.

[20] Liu M, Huang Q, Zhu Y, Chen L, Li Y, Gong Z (2022). Harnessing reactive oxygen/nitrogen species and inflammation: Nanodrugs for liver injury. Mater Today Bio.

[21] Markovic Z, Trajkovic V (2008). Biomedical potential of the reactive oxygen species generation and quenching by fullerenes (c60). Biomaterials.

[22] Gharbi N, Pressac M, Hadchouel M, Szwarc H, Wilson SR, Moussa F (2005). [60]fullerene is a powerful antioxidant in vivo with no acute or subacute toxicity. Nano Lett.

[23] Huang Y, Ren J, Qu X (2019). Nanozymes: Classification, catalytic mechanisms, activity regulation, and applications. Chem Rev.

[24] Wu J, Wang X, Wang Q, Lou Z, Li S, Zhu Y (2019). Nanomaterials with enzyme-like characteristics (nanozymes): Next-generation artificial enzymes (ii). Chem Soc Rev.

[25] Gao W, He J, Chen L, Meng X, Ma Y, Cheng L (2023). Deciphering the catalytic mechanism of superoxide dismutase activity of carbon dot nanozyme. Nat Commun.

[26] Kim M-C, Lee D, Jeong SH, Lee S-Y, Kang E (2016). Nanodiamond-gold nanocomposites with the peroxidase-like oxidative catalytic activity. ACS Appl Mater Interfaces.

[27] Zhang Y, Gao W, Ma Y, Cheng L, Zhang L, Liu Q (2023). Integrating pt nanoparticles with carbon nanodots to achieve robust cascade superoxide dismutase-catalase nanozyme for antioxidant therapy. Nano Today.

[28] Hu L, Yuan Y, Zhang L, Zhao J, Majeed S, Xu G (2013). Copper nanoclusters as peroxidase mimetics and their applications to h2o2 and glucose detection. Anal Chim Acta.

[29] Ferreira CA, Ni D, Rosenkrans ZT, Cai W (2018). Scavenging of reactive oxygen and nitrogen species with nanomaterials. Nano Res.

[30] Liu T, Xiao B, Xiang F, Tan J, Chen Z, Zhang X (2020). Ultrasmall copper-based nanoparticles for reactive oxygen species scavenging and alleviation of inflammation related diseases. Nat Commun.

[31] Wang Q-N, Duan R, Feng Z, Zhang Y, Luan P, Feng Z (2024). Understanding the synergistic catalysis in hydrogenation of carbonyl groups on cu-based catalysts. ACS Catal.

[32] Dongzhi L, Tao L, Jiang G, Chen W (2012). Synthesis of highly stable dispersions of copper nanoparticles using sodium hypophosphite. J Appl Polym Sci.

[33] Seitz HK, Bataller R, Cortez-Pinto H, Gao B, Gual A, Lackner C (2018). Alcoholic liver disease. Nat Rev Dis Primers.

[34] Fan M, Zhu C, Feng ZQ, Yang J, Liu L, Sun D (2014). Preparation of n-doped graphene by reduction of graphene oxide with mixed microbial system and its haemocompatibility. Nanoscale.

[35] Ram S, Mitra C (2001). Formation of stable cu2o nanocrystals in a new orthorhombic crystal structure. Mater Sci Eng A.

[36] Murthy HCA, Desalegn T, Kassa M, Abebe B, Assefa T (2020). Synthesis of green copper nanoparticles using medicinal plant hagenia abyssinica (brace) jf. Gmel. Leaf extract: Antimicrobial properties. J Nanomater.

[37] Huang C-L, Weng W-L, Huang Y-S, Liao C-N (2019). Enhanced photolysis stability of cu2o grown on cu nanowires with nanoscale twin boundaries. Nanoscale.

[38] Wen Y, Lambrecht J, Ju C, Tacke F (2021). Hepatic macrophages in liver homeostasis and diseases-diversity, plasticity and therapeutic opportunities. Cell Mol Immunol.

[39] Perelman A, Wachtel C, Cohen M, Haupt S, Shapiro H, Tzur A (2012). Jc-1: Alternative excitation wavelengths facilitate mitochondrial membrane potential cytometry. Cell Death Dis.

[40] Silberstein SD, Shrewsbury SB, Hoekman J (2020). Dihydroergotamine (dhe) - then and now: A narrative review. Headache.

[41] Aranda A, Sequedo L, Tolosa L, Quintas G, Burello E, Castell JV (2013). Dichloro-dihydro-fluorescein diacetate (dcfh-da) assay: A quantitative method for oxidative stress assessment of nanoparticle-treated cells. Toxicol In Vitro.

[42] Mohammad S, Thiemermann C (2020). Role of metabolic endotoxemia in systemic inflammation and potential interventions. Front Immunol.

[43] Bain CC, Mowat AM (2014). Macrophages in intestinal homeostasis and inflammation. Immunol Rev.

[44] Zhao Q, Li X, Chen G, Wang Z, Tan C, Liu C (2024). Hydrophobic nanosheet silicalite-1 zeolite for iodine and methyl iodide capture. J Hazard Mater.

[45] Wu Y, Huang T, Li X, Shen C, Ren H, Wang H (2023). Retinol dehydrogenase 10 reduction mediated retinol metabolism disorder promotes diabetic cardiomyopathy in male mice. Nat Commun.

