# Supplementary material for: Integrated cascade antioxidant nanozymes-Cu5.4O@CNDs combat acute liver injury by regulating retinol metabolism
Source: Theranostics. 2025 Apr 21;15(12):5592–615. doi: 10.7150/thno.106811 (PMC12068305; doi:10.7150/thno.106811)
Supplement: Supplementary file 1 — Supplementary materials and methods, figures and tables. [file thnov15p5592s1.pdf]

Supplementary Information

Integrated cascade antioxidant nanozymes-$Cu_{5.4}O$@CNDs combat acute liver injury by regulating retinol metabolism

Jiayu Chen[1#], Yujie Zhang[2#], Zhichao Deng[2], Yuanyuan Zhu[2], Chenxi Xu[2], Bowen Gao[2], Wenlong Wang[2], Jie Xiao[3], Zhengtao Xiao[2], Mingzhen Zhang[1, 2*], and Kangsheng Tu[1*]

Affiliations:

1. Department of Hepatobiliary Surgery, The First Affiliated Hospital of Xi'an Jiaotong University, Xi'an, Shaanxi, 710061, China

2. School of Basic Medical Sciences, Xi'an Jiaotong University, Xi'an, Shaanxi, 710061, China

3. Guangdong Provincial Key Laboratory of Nutraceuticals and Functional Foods, College of Food Science, South China Agricultural University, Guangzhou, Guangdong 510642, China

[#] These authors contribute equally to this work

* Authors for correspondence

Mingzhen Zhang (ORCID: 0000-0002-4686-6526)
E-mail address: mzhang21@xjtu.edu.cn

Kangsheng Tu (ORCID: 0000-0002-0032-1459)
Email address: tks0912@foxmail.com

Materials and methods

Materials

Activated carbon was obtained from Aladdin Chemical Reagent Co., Ltd. Sodium bicarbonate ($NaHCO_3$) was acquired from Tianli Enterprise Group Co., Ltd. Sulfuric acid (H_2SO_4) and nitric acid (HNO_3, 65%~68%) were procured from a local supplier. Cupric chloride ($CuCl_2$) was bought from Macklin (Shanghai, China). L-ascorbic acid (L-AA) and sodium hydroxide (NaOH) were obtained from Sigma Aldrich (Shanghai, China). Ferrous chloride ($FeCl_2 \cdot 4H_2O$) was obtained from Tianjin Bailens Biotechnology Co., Ltd. 3,3',5,5'-Tetramethylbenzidine (TMB) was purchased from Shanghai Malin Biochemical Technology Co., Ltd. Nitrotetrazolium blue chloride (NBT), L-methionine (L-met), and riboflavin were acquired from Sigma Aldrich. Hydrogen peroxide (H_2O_2, 30%) was procured from Tianjin Daxiong Chemical Reagent Co. Superoxide dismutase assay kit (S311) was purchased from Dojindo Molecular Technologies Co., Ltd. Hydrogen Peroxide Assay Kit, Total Antioxidant Capacity Assay Kit with ABTS method, 2',7'-dichlorodihydrofluorescein diacetate (DCFH-DA), dihydroethidium (DHE) and animal RNA isolation kits were purchased from Beyotime Chemical Reagent Co., Ltd. 4', 6-diamidino-2-pheny-lindole (DAPI) was obtained from Roche Applied Science. All chemical reagents were not further purified and were used directly, and detection kits were used according to the manufacturer's instructions. All aqueous solutions used in this work were prepared with deionized water with a resistivity of 18.2 MΩ·cm.

Instrumentation

Transmission electron microscopy (TEM) images were obtained using a FEI Tecnai G2 F30 (FEI, USA) at an acceleration voltage of 300 kV. Powder X-ray diffraction (XRD) data were collected using a Bruker D8 ADVANCE (Germany) with a 6 ° / min scan rate. FT-IR spectra were recorded by a Thermo

Fisher Nicolet 5700 (USA). X-ray photoelectron spectroscopy (XPS) spectra were recorded by a Thermo Escalab 250Xi (USA). Electron spin resonance (ESR) spectra were recorded by a Bruker A300-9.5/12 (Switzerland) at room temperature. Flow cytometry data were collected by FACS CaliburTM, Becton Dickinson (USA). Microplate absorbance was measured using a Tecan Spark 20 M multi-mode microplate reader (Switzerland). The fluorescence imaging was done using an *in vivo* imaging instrument (IVIS Lumina 3, PE, USA), and images were obtained by IVIS Living Image 3.0 software (PerkinElmer, USA). Leica DM3000 microscope (Leica, Wetzlar, Germany) was used to image tissue sections.

Synthesis of CNDs-A or Re-CNDs-A

The CNDs-A or Re-CNDs-A were synthesized according to the methodology reported previously [1]. 20 mg of CNDs was added to 50 ml of $NaBH_4$ solution (0.5 M), and the mixture was stirred for 24 h at room temperature. The resulting solution was neutralized with hydrochloric acid and further dialyzed for 3 days to obtain Re-CNDs.

Then, 1 mL of CNDs or Re-CNDs solution (5 mg/mL) and 1g of 1,3-propanesultone (PS) were added to 10 mL of 1, 4-dioxane. A total of 1 mL triethylamine was then added to this mixture. After stirring for 24 h at 40 °C, the mixture underwent rotary evaporation to remove the solvent. The resulting sample was dispersed in water and dialyzed in 0.1 M NaCl solution for 1 day to remove triethylamine salt through ion exchange and then dialyzed in ultrapure water for 3 days.

5 mg of the resulting CNDs or Re-CNDs (reacted with PS) was added to 10 mL of 0.5 M NaOH solution and then stirred for 24 h at 40 °C. The resulting solution was neutralized with hydrochloric acid and then dialyzed for 3 days to obtain CNDs-A or Re-CNDs-A.

Synthesis of CNDs-B

The CNDs-B were synthesized according to the methodology reported previously [1].5 mg of CNDs was heated with 2-3 mL of thionyl chloride ($SOCl_2$) in 6-8 mL of acetonitrile until all the CNDs solid powder dispersed in the solution. After removing the excess $SOCl_2$ and acetonitrile, the CNDs acyl chloride was obtained. The CNDs acyl chloride was redispersed in 6-8 mL of anhydrous acetonitrile with N-Boc-ethylenediamine (200 μL) under stirring for 1h at room temperature. After removing the excess solvent, 7.5 mL of dichloromethane (CH_2Cl_2) and 2.5 mL of trifluoroacetic acid (CF_3COOH) were added to the sample. After stirring for 12 h, remove the excess solvent and add 2-3 mL of water. The resulting solution was further dialyzed in ultrapure water for 2-3 days.

1 mL of the resulting CNDs solution (5 mg/mL) and 1 g of 1,3-propanesultone (PS) were added to 10 mL of 1, 4-dioxane. A total of 1 mL triethylamine was then added to this mixture. After stirring for 24 h at 40 °C, the mixture underwent rotary evaporation to remove the solvent. The resulting sample was dispersed in water and dialyzed in 0.1 M NaCl solution for 1 day to remove triethylamine salt through ion exchange and then dialyzed in ultrapure water for 3 days.

Synthesis of CNDs-Cy5.5

5 mg of CNDs was heated with 2-3 mL of thionyl chloride ($SOCl_2$) in 6-8 mL of acetonitrile until all the CNDs solid powder dispersed in the solution. After removing the excess $SOCl_2$ and acetonitrile, the CNDs acyl chloride was obtained. The CNDs acyl chloride was redispersed in 6-8 mL of anhydrous acetonitrile with Sulfo-Cyanine5.5 amine (3 mg/mL, 167 μL) under stirring for 30 min at room temperature in the dark. After that, the mixture underwent rotary evaporation to remove the solvent. The resulting sample was dispersed in water and stirred for 1-2 h. After then, the resulting solution was filtrated with PD-10 Columns several times to obtain the CNDs-Cy5.5.

Animals

Female BALB/c mice (6-8 w, 20-25 g) were purchased from Xi’an Jiaotong University, Xi’an, Shaanxi Province, China. All mice were kept under standard conditions, including light, temperature, water, and food, and animal experiments were approved by the Animal Ethics Committee of Xi'an Jiaotong University.

Transcriptome analysis

HIRI and LPS-ALI model mice were divided into three groups: Con, modeling group, and treatment group, respectively. Liver tissues were taken from the mice after execution at the end of the experiment and stored on ice. Total RNA was extracted from the liver tissue using Trizol reagent according to the manufacturer's protocol, and the purity and concentration of the RNA were evaluated by a NanoDrop 2000 spectrophotometer. Only high-quality RNA samples were used for the construction of sequencing libraries ($OD_{260/280}$ = 1.8-2.2, RIN ≥ 6.5, 28S: 18S ≥ 1.0, > 2 μg). The mRNA with PolyA (polyadenylate) in it was captured explicitly by two rounds of purification using oligo(dT) magnetic beads (Dynabeads Oligo (dT), cat.25-61005, Thermo Fisher, USA). The captured mRNAs were fragmented using the Magnesium Ion Interruption Kit (NEBNextR Magnesium RNA Fragmentation Module, cat. E6150S, USA) at high temperature, 94 °C, 5-7 min. To synthesize cDNA, the fragmented RNA was passed through reverse transcriptase (Invitrogen SuperScriptTM II Reverse Transcriptase, cat.1896649, CA, USA). cDNA was then synthesized using E. coli DNA polymerase I (NEB, cat.m0209, USA) with RNase H (cat.m0209, USA). USA) and RNase H (NEB, cat.m0297, USA) for two-stranded synthesis to convert these composite double strands of DNA and RNA into DNA duplexes, while the duplexes were doped with dUTP Solution (Thermo Fisher, cat.R0133, CA, USA) to make up the ends of the double-stranded DNA to flat ends, and then An A base was added to each

of its ends to enable it to be ligated to a junction with a T base at the end, and its fragment size was screened and purified using magnetic beads. The second strand was digested with UDG enzyme (NEB, cat.m0280, MA, US), and the fragment size of 300 bp ± 50 bp was formed by PCR-pre-denaturation holding at 95 °C for 3 min, denaturation at 98 °C for a total of 8 cycles of 15 s each, annealing to 60 °C holding at 60°C for 15 s, extension at 72 °C for 30 s, and a final extension held at 72 °C for 5 min. 50 bp library (strand-specific library). Finally, the library was bipartite sequenced using Illumina NovaseqTM 6000 (LC Bio-Technology CO., Ltd. Hangzhou, China) according to the standard operation, and the sequencing mode was PE150.

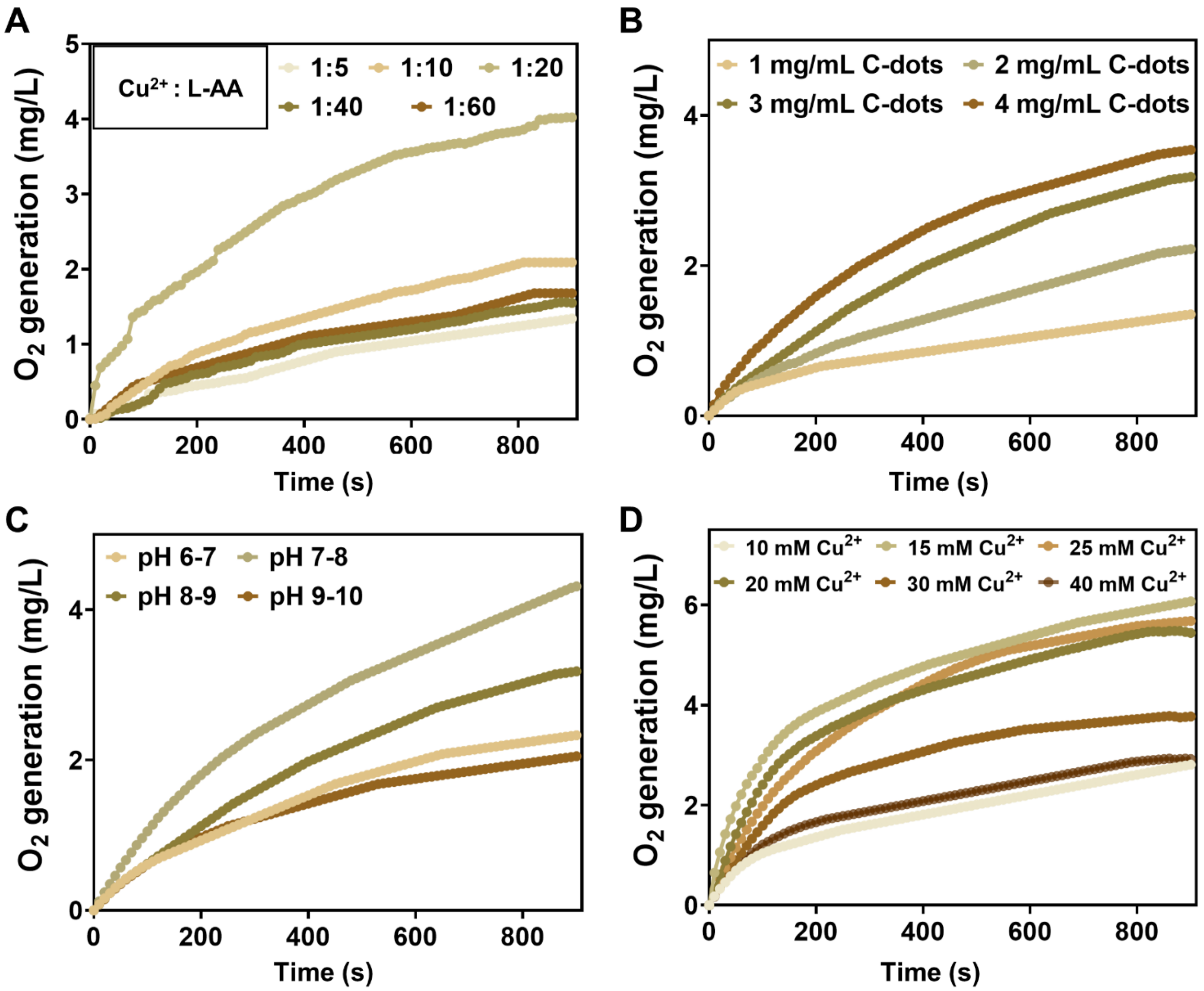

Supplementary Figure 1. Different synthesis conditions for $Cu_{5.4}O$ USNPs, $Cu_{5.4}O$@CNDs. (A) Dissolved oxygen assay of hydrogen peroxide scavenging capacity of $Cu_{5.4}O$ USNPs synthesized by Cu^{2+}: L-AA with different feeding ratios (1:5, 1:10, 1:20, 1:40, 1:60). (B) Dissolved oxygen assay of hydrogen peroxide scavenging capacity of $Cu_{5.4}O$@CNDs synthesized by C-dots with different feeding ratios (1-4 mg/mL). (C) Dissolved oxygen assay of hydrogen peroxide scavenging capacity of synthesized $Cu_{5.4}O$@CNDs at different pH (pH 6-7, 7-8, 8-9, 9-10). (D) Dissolved oxygen assay of hydrogen peroxide scavenging capacity of synthesized $Cu_{5.4}O$@CNDs at different concentrations of Cu^{2+}.

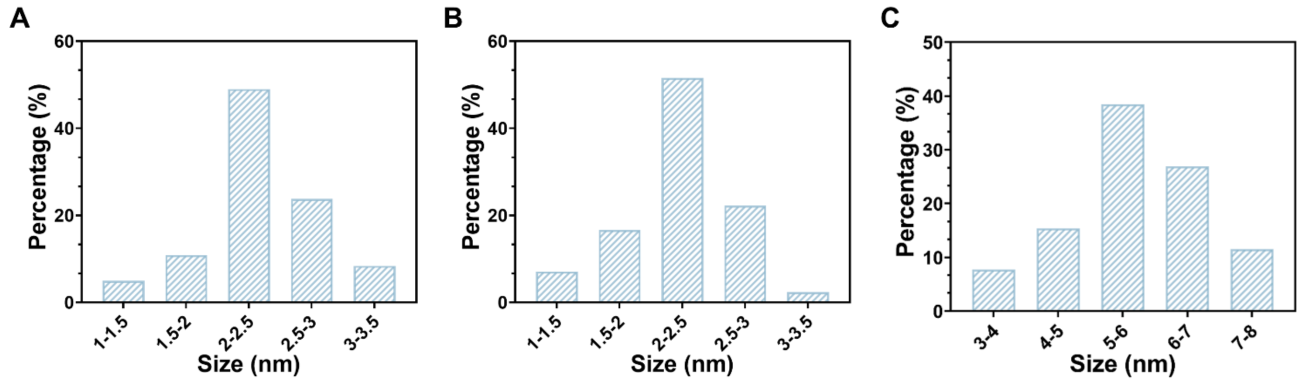

Supplementary Figure 2. Characterization of C-dots, $Cu_{5.4}O$ USNPs, $Cu_{5.4}O$@CNDs. (A) Particle size statistics of C-dots. (B) Particle size statistics of $Cu_{5.4}O$ USNPs. (C) Particle size statistics of $Cu_{5.4}O$@CNDs.

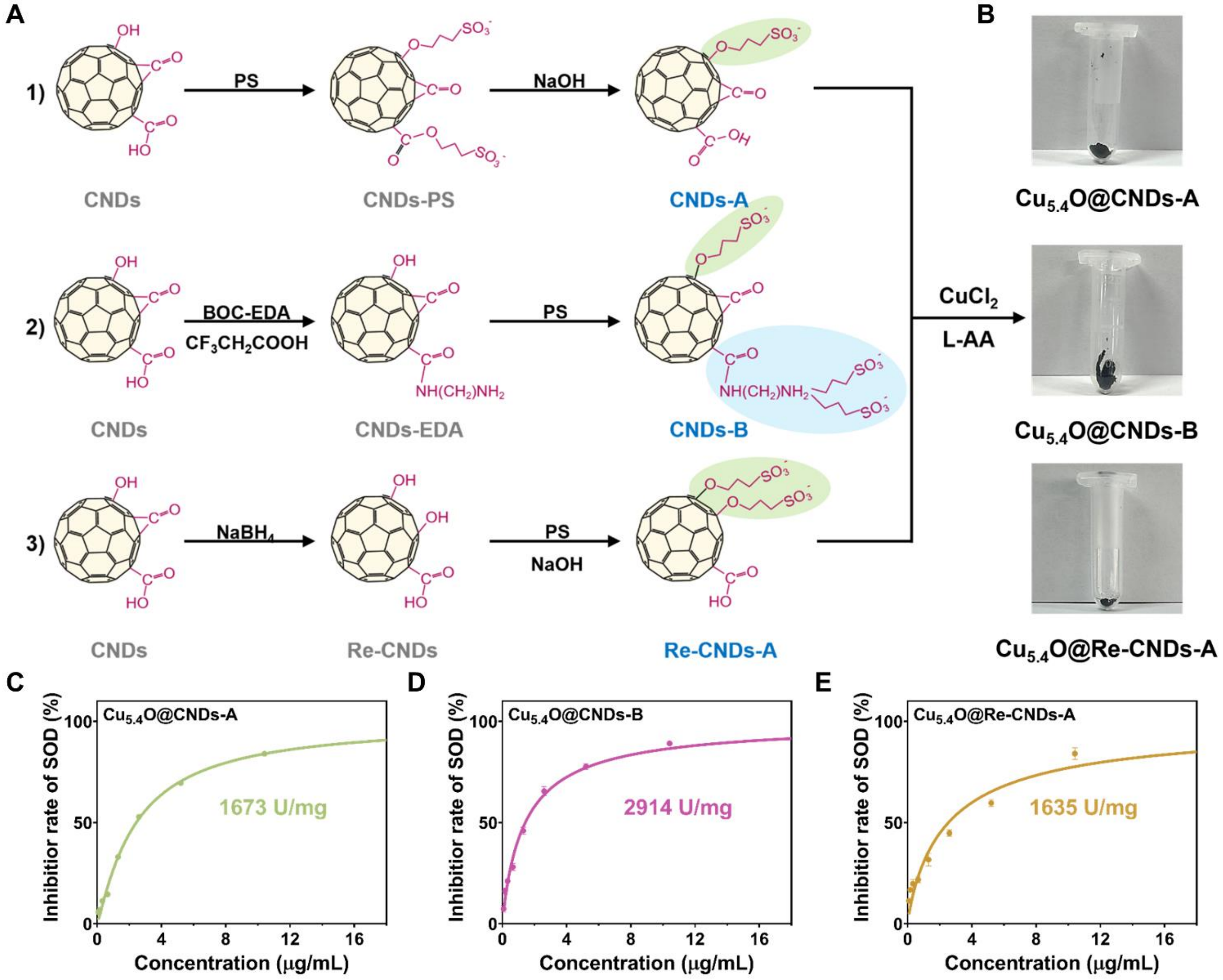

Supplementary Figure 3. Surface structure modification to study the mechanism of synergy effect between $Cu_{5.4}O$ USNPs and CNDs. (A) Illustration of CNDs modification. (B) Picture of the synthesized samples of $Cu_{5.4}O$@CNDs-A, $Cu_{5.4}O$@CNDs-B and $Cu_{5.4}O$@Re-CNDs-A. (C-E) The SOD-like activities of $Cu_{5.4}O$@CNDs-A, $Cu_{5.4}O$@CNDs-B and $Cu_{5.4}O$@Re-CNDs-A.

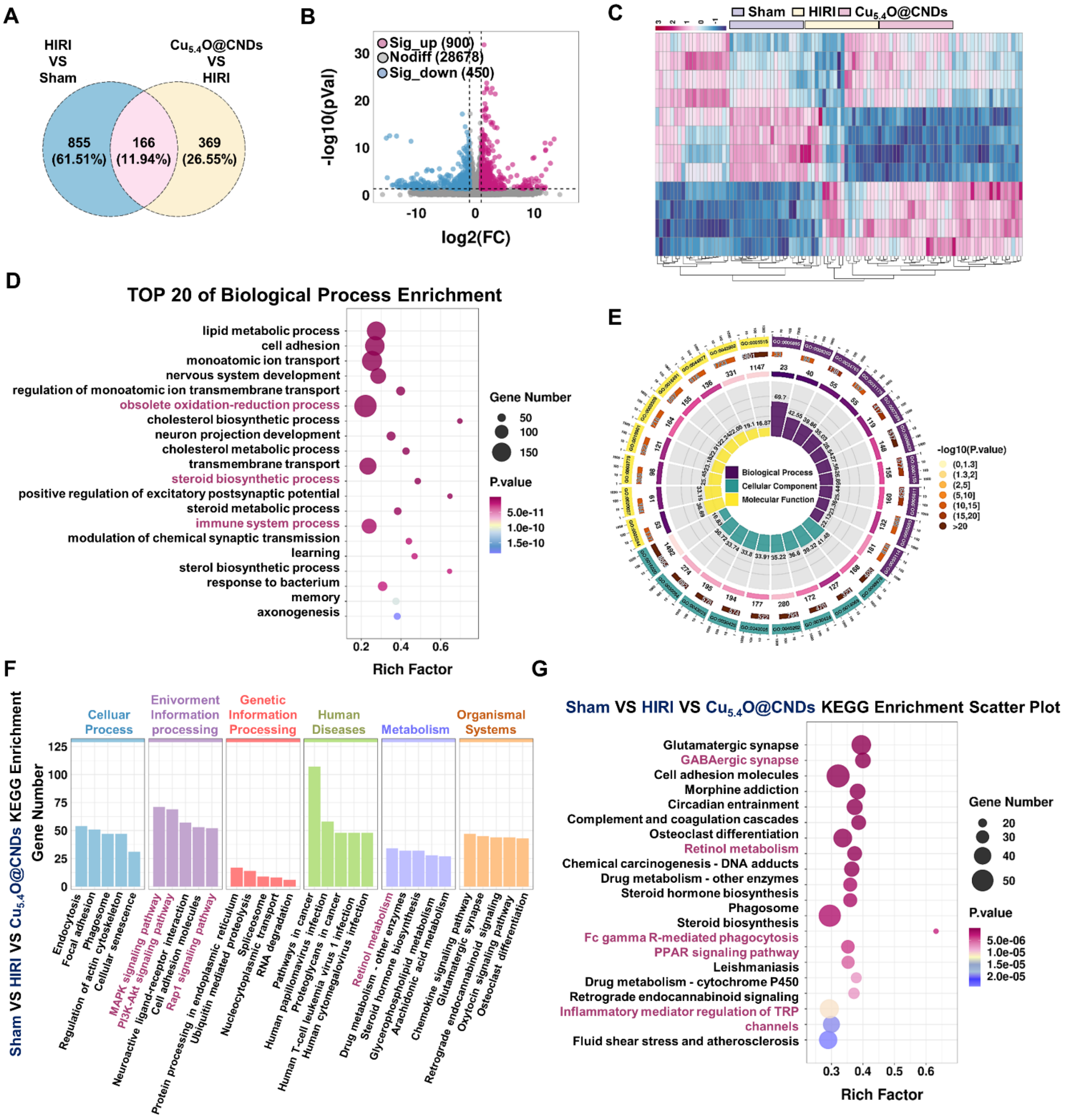

Supplementary Figure 4. Therapeutic mechanisms of $Cu_{5.4}O$@CNDs on HIRI. (A) Venn diagram of whole transcriptome RNA sequencing analysis showed differences in the expression of genes in the HIRI group VS the control group and genes in the $Cu_{5.4}O$@CNDs group VS the HIRI group. (B) Volcano plots showed the identified upregulated and downregulated genes by $Cu_{5.4}O$@CNDs. (C) Heat maps of significant genes after $Cu_{5.4}O$@CNDs treatment (fold change≥2 and P< 0.01). (D) GO analysis. The 20 most significantly enriched Biological Processes were shown. (E) GO analysis. (Molecular Function, MF; Biological Process, BP; Cellular Components, CC). (F) KEGG analysis. (G) KEGG pathway enrichment analysis. The 20 most significantly enriched pathways were shown.

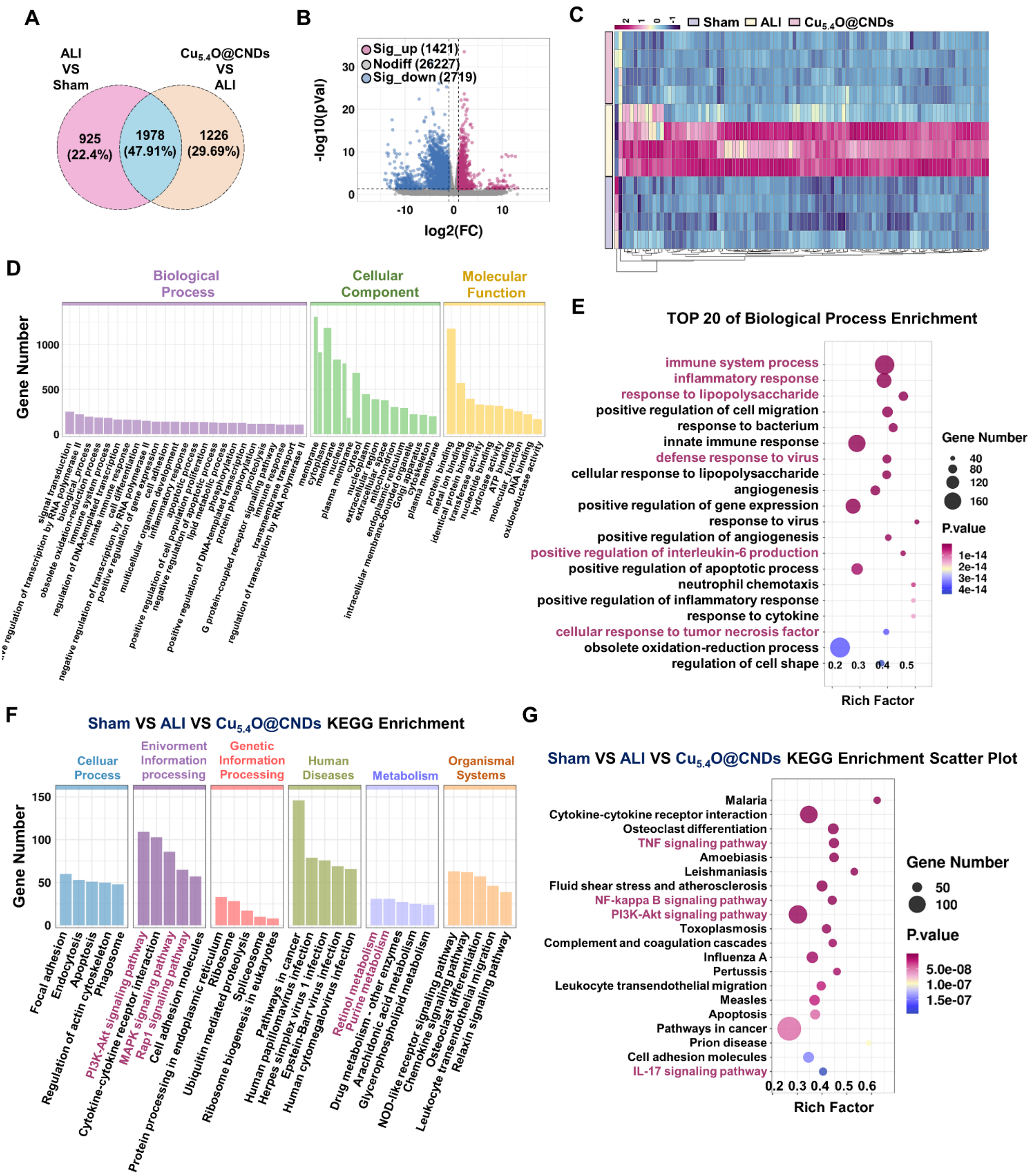

Supplementary Figure 5. Therapeutic mechanisms of $Cu_{5.4}O$@CNDs on LPS-ALI. (A) Venn diagram of whole transcriptome RNA sequencing analysis showed differences in the expression of genes in the ALI group VS the control group and genes in the $Cu_{5.4}O$@CNDs group VS the ALI group. (B) Volcano plots showed the identified upregulated and downregulated genes by $Cu_{5.4}O$@CNDs. (C) Heat maps of significant genes after $Cu_{5.4}O$@CNDs treatment (fold change≥2 and P< 0.01). (D) GO analysis. (Molecular Function, MF; Biological Process, BP; Cellular Components, CC). (E) GO analysis. The 20 most significantly enriched Biological Processes were shown. (F) KEGG analysis. (G) KEGG pathway enrichment analysis. The 20 most significantly enriched pathways were shown.

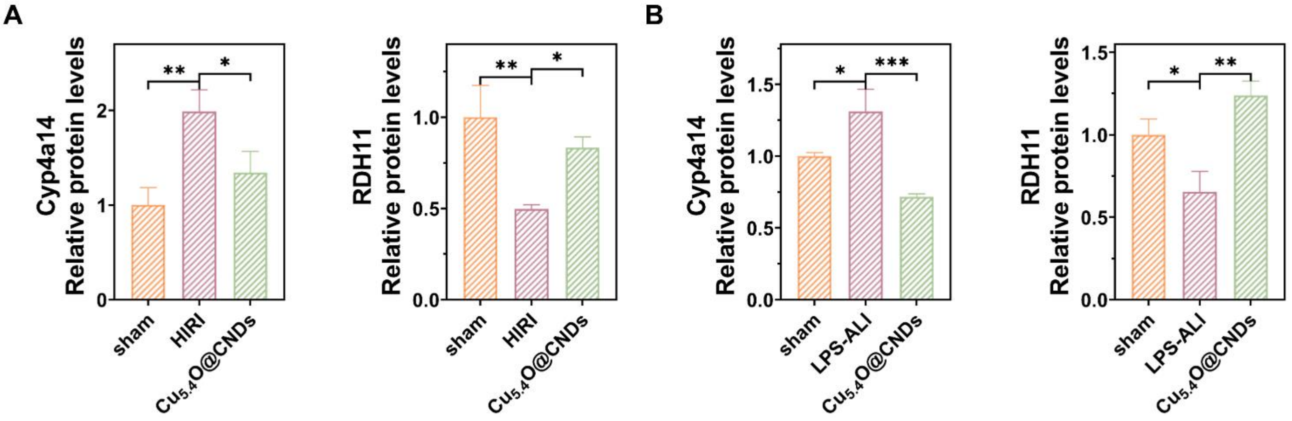

Supplementary Figure 6. Quantification of hepatic protein expression of CYP4a14 and RDH11. (A) Quantification of protein expression of CYP4a14 and RDH11 in the HIRI liver model (n=3). (B) Quantification of protein expression of CYP4a14 and RDH11 in the LPS-ALI liver model (n=3).

Supplementary Table 1. Sequences of the primers used for qRT-PCR.

Gene	Forward sequences	Reverse sequences
Mouse IL-1β	5-TCGCTCAGGGTCACAAGAAA-3	5-CATCAGAGGCAAGGAGGAAAAC-3
Mouse IL-6	5-ACAAGTCGGAGGCTTAATACACAT-3	5-TTGCCATTGCACAACTCTTTTC-3
Mouse IL-12	5-GCCAGTACACCTGCCACAAAF-3	5-TGTGGAGCAGCAGATGTGAGT-3
Mouse TNF-α	5-AGGCTGCCCCGACTACGT-3	5-GACTTTCTCCTGGTATGAGATAGCAAA-3
Mouse GAPDH	5-TGTGTCCGTCGTGGATCTGA-3	5-TTGCTGTTGAAGTCGCAGGAG-3
Human IL-1β	5-CTCTCCACCTCCAGGGACAGG-3	5-TCAACACGCAGGACAGGTACAG-3
Human IL-6	5-TGGTGTTGCCTGCTGCCTTC-3	5-GCTGAGATGCCGTCGAGGATG-3
Human IL-12	5-GACCTTGGACCAGAGCAGTGAG-3	5-TGAAGCAGCAGGAGCGAATGG-3
Human TNF-α	5-GGCGTGGAGCTGAGAGATAACC-3	5-ACGGCGATGCGGCTGATG-3
Human GAPDH	5-CACCCACTCCTCCACCTTTGAC-3	5-GTCCACCACCCTGTTGCTGTAG-3

Supplementary Table 2. The primary antibodies were used for immunoblot.

Antibody	Host Animal	Dilution for Immunoblot	Distributor	Cat. Num.
Vinculin	Mouse	1:1000	Santa Cruz Biotechnology	sc-73614
CYP4a14	Rabbit	1:1000	Abways	CY8721
RDH11	Rabbit	1:1000	Immunoway	YN7538

Reference

1. Zhang Y, Gao W, Ma Y, Cheng L, Zhang L, Liu Q, et al. Integrating pt nanoparticles with carbon nanodots to achieve robust cascade superoxide dismutase-catalase nanozyme for antioxidant therapy. Nano Today. 2023; **49**.
